# Dexketoprofen/tramadol: randomised double-blind trial and confirmation of empirical theory of combination analgesics in acute pain

**DOI:** 10.1186/s10194-015-0541-5

**Published:** 2015-06-27

**Authors:** R. Andrew Moore, C. Gay-Escoda, R. Figueiredo, Z. Tóth-Bagi, T. Dietrich, S. Milleri, D. Torres-Lagares, C. M. Hill, A. García-García, P. Coulthard, A. Wojtowicz, D. Matenko, M. Peñarrocha-Diago, S. Cuadripani, B. Pizà-Vallespir, C. Guerrero-Bayón, M. Bertolotti, M. P. Contini, S. Scartoni, A. Nizzardo, A. Capriati, C. A. Maggi

**Affiliations:** Pain Research and Nuffield Division of Anaesthetics, Nuffield Department of Clinical Neurology, University of Oxford, The Churchill, Oxford, OX3 7LE UK; Department of Oral and Maxillofacial Surgery, School of Dentistry, University of Barcelona, Bellvitge Institute for Biomedical Research (IDIBELL), Barcelona, Spain; Dr. Tóth Bagi Zoltán Fogászati Rendeloje, Budapest, Hungary; The School of Dentistry, College of Medical and Dental Sciences, University of Birmingham, Birmingham, UK; Centro Ricerche Cliniche, Ospedale Policlinico G.B.Rossi, Verona, Italy; Hospital Universitario Virgen del Rocío, Departamento de Estomatología, Facultad de Odontología, Universidad de Sevilla, Sevilla, Spain; University Dental Hospital, Cardiff, UK; Department of Maxillofacial Surgery, Hospital Médico Quirúrgico de Conxo, Santiago de Compostela, Spain; School of Dentistry, University of Manchester, Manchester, UK; Department of Oral Surgery, Warsaw Medical Univeristy, Warsaw, Poland; Departament d’Estomatologia, Clínica Odontológica, Universidad de Valencia, Valencia, Spain; Clinical Research, Laboratorios Menarini S.A. – Menarini Group, Badalona, Spain; Clinical Research, Menarini Ricerche S.p.A – Menarini Group, Florence, Italy

**Keywords:** Dexketoprofen, Tramadol, Combination analgesics, Postoperative pain, Third molar, Randomised controlled trial, Dose range

## Abstract

**Background:**

Combination analgesics are effective in acute pain, and a theoretical framework predicts efficacy for combinations. The combination of dexketoprofen and tramadol is untested, but predicted to be highly effective.

**Methods:**

This was a randomised, double-blind, double-dummy, parallel-group, placebo-controlled, single-dose trial in patients with moderate or severe pain following third molar extraction. There were ten treatment arms, including dexketoprofen trometamol (12.5 mg and 25 mg) and tramadol hydrochloride (37.5 mg and 75 mg), given as four different fixed combinations and single components, with ibuprofen 400 mg as active control as well as a placebo control. The study objective was to evaluate the superior analgesic efficacy and safety of each combination and each single agent versus placebo. The primary outcome was the proportion of patients with at least 50 % max TOTPAR over six hours.

**Results:**

606 patients were randomised and provided at least one post-dose assessment. All combinations were significantly better than placebo. The highest percentage of responders (72 %) was achieved in the dexketoprofen trometamol 25 mg plus tramadol hydrochloride 75 mg group (NNT 1.6, 95 % confidence interval 1.3 to 2.1). Addition of tramadol to dexketoprofen resulted in greater peak pain relief and greater pain relief over the longer term, particularly at times longer than six hours (median duration of 8.1 h). Adverse events were unremarkable.

**Conclusions:**

Dexketoprofen trometamol 25 mg combined with tramadol hydrochloride 75 mg provided good analgesia with rapid onset and long duration in a model of moderate to severe pain. The results of the dose finding study are consistent with pre-trial calculations based on empirical formulae.

**Trial registration:**

EudraCT (2010-022798-32); Clinicaltrials.gov (NCT01307020).

**Electronic supplementary material:**

The online version of this article (doi:10.1186/s10194-015-0541-5) contains supplementary material, which is available to authorized users.

## Background

Greater efficacy from combination analgesics in acute pain has been recognised for some time [1], albeit originally in cancer pain. When oral analgesic drugs are tested in standard, acute pain models [2] those with the highest efficacy and lowest numbers-needed-to-treat (NNT) are typically high doses of individual analgesics or low doses of combinations of analgesics [3]. Combinations of drugs of high efficacy include paracetamol and codeine [4], paracetamol and oxycodone [5], ibuprofen and codeine [6], ibuprofen and oxycodone [7], and ibuprofen and paracetamol [8]. Even adding caffeine can improve analgesic efficacy as a combination with conventional analgesics [9].

The efficacy of combination analgesics has been shown to be the sum of the efficacies of the individual analgesic components, and was broadly true across a range of different drug combinations, in postoperative pain and migraine headache, and when tested in the same and different trials [10]. This means that the efficacy of any proposed combination can be assessed theoretically before clinical trials are conducted.

A potential part of any combination might be a fast-acting non-steroidal anti-inflammatory drug (NSAID) formulation, because speed of absorption and onset produces good and long lasting analgesia [11, 12]. Dexketoprofen is effective in acute pain at low doses [13], and is also effective in a wide variety of pain conditions [14]; dexketoprofen is the active chiral form of ketoprofen. Tramadol is a widely used opioid of proven efficacy in combination with paracetamol [15].

This study therefore aimed at evaluating the superior analgesic efficacy and safety of dexketoprofen trometamol and tramadol hydrochloride given as four different fixed combinations and as single components in comparison to placebo, on moderate to severe acute pain following impacted third mandibular molar tooth extraction. It was also intended to select the optimum dose combination(s) to be further evaluated in the subsequent phase III pivotal studies.

We used a formula derived empirically to estimate the possible efficacy that might be obtained from dexketoprofen and tramadol dosing combinations [10]. There were limited data for dexketoprofen from a systematic review [13] and two individual patient level analyses of tramadol [16, 17]. Estimated NNTs for combinations with dexketoprofen trometamol 25 mg and tramadol hydrochloride 37.5 mg or 75 mg were 2.2 and 1.6, respectively. Estimates for combinations with lower dexketoprofen trometamol doses (12.5 mg) were higher (worse) than 3 or above. There was limited confidence in the NNT estimates for the combinations due to uncertainty in the efficacy estimates of individual drugs because of low numbers.

## Methods

The study (Sponsor Code DEX-TRA-02; EudraCT number 2010-022798-32) was registered at clinicaltrials.gov (NCT01307020). It was performed at 16 study sites in six European countries (Germany, Hungary, Italy, Poland, Spain and the United Kingdom). It was conducted in accordance with the principles of Good Clinical Practice and the Declaration of Helsinki and was approved by all the concerned Competent Authorities and Ethics Committees. All participating patients provided written informed consent. The clinical phase of the study started on 23rd February 2011 (first patient screened) and concluded on 14th October 2011 (last patient out).

### Patients

Healthy male or female patients, aged 18 to 70 years, were eligible for the study if they were scheduled for outpatient surgical removal, under local anaesthesia, of one or more third molars, at least one of which was fully or partially impacted in mandibular bone. Criteria for randomisation included postoperative pain of moderate to severe intensity (Visual Analogue Scale [VAS] ≥40 mm and 4-point Verbal Rating Scale [VRS] ≥2) within four hours after surgery.

Patients were excluded from the study in any of the following circumstances: pregnant or breastfeeding women or women of child-bearing potential not using adequate contraception; known allergy to the study drugs, paracetamol, acetylsalicylic acid, opioids or other NSAIDs; moderate to severe renal, hepatic or cardiac dysfunction; history of gastrointestinal disorders, bleeding disorders; epilepsy, asthma, angioedema or related disorders; history of drug or alcohol abuse; presence of any medical condition that in the opinion of the investigator might pose a risk to the patient, may confound study results or might impair compliance with the study procedures. Patients who had received any investigational drug or participated in any other clinical trial within the previous month were also excluded. Further exclusion criteria included significant surgical complications, overall surgery duration longer than one hour and need for re-anaesthesia. Patients who had taken any analgesics less than 24 h before surgery were also excluded. Concomitant use of alcohol, psychoactive drugs, sedatives and any other medications or therapies that could pose a risk to the patient or confound the study results were not permitted within 48 h and two weeks before surgery (depending on the half life of the respective medications) and up to 24 h post-dose. Local application of ice and the intake of caffeine were not permitted during the 24-hour post-dose period. Patients had to be in fasting conditions from two hours before surgery and up to three hours post-dose.

### Study design

This was a multicentre, randomised, double-blind, double-dummy, parallel-group, placebo-controlled, single-dose, phase II, dose-finding study, with a total of 10 treatment arms (with balanced allocation ratio), including dexketoprofen trometamol (12.5 mg and 25 mg) and tramadol hydrochloride (37.5 mg and 75 mg) given as four different fixed combinations (DKP12.5/TRAM37.5; DKP12.5/TRAM75; DKP25/TRAM37.5; DKP25/TRAM75) and as single components (DKP12.5; DKP25; TRAM37.5; TRAM75). An active control (ibuprofen 400 mg, as an acid formulation) was included in order to demonstrate the sensitivity of the pain model, because it was significantly superior to placebo in trials in the same indication [18–20], and has the largest body of data for the indication [3].

The overall study duration was approximately 30 days for each patient, including three visits to the study site: Visit 1, for screening (within two weeks of their scheduled surgery day); Visit 2, for dental surgery, randomisation and treatment administration, followed by a 24-hour post-dose pain and analgesic effect assessment period (with the first four hours at the study site), during which patients had to record efficacy data using an electronic diary (eDiary); and Visit 3 (End of Study), for final safety follow-up (10 ± 3 days after surgery day). In addition, patients received a phone call for safety assessment the day after Visit 2 (approximately 24 h post-dose).

For those patients who met the selection criteria, the surgical procedure was performed under standardised local anaesthesia, which was limited to local anaesthetic block using lidocaine (2 %) with epinephrine (1:80.000) up to a total volume of 5.4 mL per molar. After surgery, patients reporting pain were asked to rate their pain intensity (PI) by a VAS (0–100; with the left end labelled “no pain” and the right end labelled “worst possible pain”; [21, 22]) and by a 4-point VRS (0 = none, 1 = mild, 2 = moderate, 3 = severe; [21]) to assess their eligibility for randomisation. Patients experiencing pain of moderate to severe intensity (VAS ≥40 mm and VRS ≥ 2) within four hours after the end of surgery were randomised and received one single oral dose of the assigned study treatment. After randomisation and immediately prior to the administration of study medication, VRS-PI was measured again and the score was recorded as baseline PI for the efficacy analysis.

Participants were randomly assigned to one of 10 treatment groups following a blocked randomisation procedure, with a block size of 10 and an allocation ratio of 1:1:1:1:1:1:1:1:1:1. The randomisation process was centralised by an Interactive Voice/Web Response System (IVRS/IWRS) and the treatment code was delivered for each patient according to a computer-generated random allocated sequence (randomisation list) prepared by a Sponsor’s third party prior to the start of the study. Two sets were prepared, one set was used for programming the IVRS/IWRS and the other set was used for the labelling of the study medication. Personnel involved in the preparation or the handling of the randomisation list were not involved in the study conduct and statistical analysis. Double-blind conditions were secured by the identical appearance and weight of the eight tested study drugs as tablets as well as the placebo tablet matching the tested study drugs. In order to keep the active control ibuprofen blinded, there was also a placebo tablet matching ibuprofen (each single-dose treatment consisted of two tablets) leading to a double-dummy design. The blind was maintained for patients and for people responsible for the ongoing conduct of the study (such as the management, monitors, investigators) and those responsible for data analysis and interpretation of results at the conclusion of the study, such as biometrics personnel.

Rescue medication (RM) consisting of paracetamol 1 g (with a maximum recommended daily dose of 4 g) was available on request during the 24-hour post-dose period. Patients were encouraged but not compelled to wait for at least 60 min post-dose, to allow time for the study medication to take effect.

### Efficacy evaluation

Following treatment administration, patients were requested to make multiple assessments of pain intensity and pain relief (PAR) on the eDiary over a period of 24 h. They also had to make an overall assessment of the study medication (patient global evaluation, PGE) at the end of this period. The time when RM was first used, if applicable, was also recorded.

PI was measured on a 4-point VRS (0 = none, 1 = mild, 2 = moderate, 3 = severe; [21]) immediately prior the administration of study medication (baseline PI) and then at 15 min, 30 min, 45 min, 1 h, 1.5 h, 2 h, 2.5 h, 3 h, 3.5 h, 4 h, 5 h, 6 h, 8 h, 12 h and 24 h post-dose. PAR was measured on a 5-point VRS (0 = none, 1 = slight, 2 = moderate, 3 = good, 4 = complete; [21]) at the same pre-defined post-dose time points. PGE was measured on a 5-point VRS (1 = poor, 2 = fair, 3 = good, 4 = very good, 5 = excellent; [21, 23]) at 24 h post-dose (or whenever the patient used RM, if this occurred first). When patients used RM, a final PI and PAR assessment and the PGE were recorded immediately before the intake and, after that, they were excluded from further efficacy measurements. After use of RM, the *baseline observation carried forward (BOCF) method* was applied [24], with PI returning to its baseline score and PAR to zero for all subsequent time points. If a patient prematurely withdrew from the study, final PI and PAR assessment and the PGE were also requested.

From the PI and PAR scores, the summed pain intensity differences (SPID) and the total pain relief (TOTPAR) over 4, 6, 8 and 12 h post-dose were calculated. SPID was calculated as the time-weighted sum of the pain intensity difference (PID) values from baseline and TOTPAR was calculated as the time-weighted sum of the PAR scores. The percentages of the theoretical maximum possible SPID (% max SPID) and of the theoretical maximum possible TOTPAR (% max TOTPAR) were also calculated.

The primary efficacy endpoint was the percentage of patients showing response, defined as the achievement of at least 50 % of the maximum possible TOTPAR (≥50 % max TOTPAR), over 6 h post-dose within the respective treatment arm [3, 24].

Secondary efficacy endpoints included: percentage of responders (≥50 % max TOTPAR) over 4, 8 and 12 h; mean PI and PAR (VRS) scores over 24 h; SPID, % max SPID, TOTPAR and % max TOTPAR over 4, 6, 8 and 12 h; PGE at the end of the assessment period; time to first use of RM since treatment administration and percentage of patients using RM over 4, 6, 8, 12 and 24 h.

### Safety evaluation

The safety evaluation was based on the incidence, seriousness, intensity and causal relationship of treatment-emergent adverse events (AEs). AEs were assessed throughout the entire study by means of a non-leading open question. Spontaneously reported AEs were also recorded. Furthermore, safety was also evaluated by the assessment of clinically significant changes post-dose versus baseline in physical examination, vital signs (VS; blood pressure and heart rate), 12-lead electrocardiogram (ECG) and laboratory safety tests (haematology, biochemistry and urinalysis). Any patient who prematurely withdrew after having received study medication was encouraged to undergo Visit 3.

### Statistical analysis

For the primary efficacy variable, in order to demonstrate the superiority of active treatment in comparison with placebo the null hypothesis of equality between placebo and each tested study drug (the four combinations and the four corresponding single agents) was tested using a Chi-square test. Multiplicity was adjusted by using the Šidák correction [α =1-(1- α) ^ (1/k)], where k was the number of comparisons. Considering eight comparisons, a type I error probability = 0.00639 was used for the single comparisons. The null hypothesis of equality between placebo and the active control was also tested to validate the pain model. In addition, event rates (ER), relative risk (RR), NNT and relative risk reduction (RRR), with their corresponding 95 % confidence intervals (CI), were estimated in order to compare the effect size of placebo with the effect size of each active treatment.

Secondary efficacy variables were analysed as follows: percentage of responders over 4, 8 and 12 h were analysed analogously to the primary efficacy variable; mean PI and PAR (VRS) scores were analysed by means of descriptive statistics; quantitative variables (TOTPAR, % max TOTPAR, SPID and % max SPID) showing homogeneity of variance (according to Levene’s test) were analysed by one-way analysis of variance (ANOVA) using the Dunnett’s test for comparison between placebo and each active treatment, with an overall significance level of 5 % two-sided; ordinal variables (PGE) and quantitative variables showing no homogeneity of variance were analysed by the Wilcoxon rank-sum test for comparison between placebo and each active treatment, using the Hochberg correction for the adjustment for multiple comparisons, with an overall significance level of 5 % two-sided; time to RM was analysed using the Kaplan-Meier estimation method and treatment groups were compared using a log-rank test, with the Hochberg method applied for the multiplicity correction; percentage of patients using RM were analysed analogously to the primary efficacy variable. Safety variables were analysed by means of descriptive statistics.

As an exploratory analysis, the primary efficacy endpoint was reassessed using active control as comparator. The f statistics test was used to evaluate if the effect of dexketoprofen, tramadol and their combination was statistically significant on the outcome. When at least one of the three f tests was found to be statistically significant, the Tukey method was applied to find out which doses gave a significant difference on the outcome. Statistical differences between NNTs were examined using the z-test [25].

All efficacy analyses were performed on the “intention-to-treat” (ITT) population (randomised patients who received study medication and for whom at least one post-dose assessment was available). The “per protocol” (PP) population (patients of the ITT population with no major protocol violations) was used to perform confirmatory analyses on the primary endpoint. Safety analyses were performed on the “safety” population (randomised patients who received study medication).

It was estimated that 540 evaluable patients (60 per treatment arm) would have to be included in order to achieve approximately 80 % power in rejecting the null hypothesis of equality between placebo and the eight experimental treatment arms (the four combinations and the four corresponding single agents) regarding the primary endpoint, on the basis of the following assumptions [26]: response rate for placebo = 0.13; expected RR of response in active treatment versus placebo = 3.21; overall type I error probability of 0.05 (two-sided). Sixty further patients were to be treated with the active control as a reference for the effect of the eight experimental treatment arms. It was expected that approximately 667 patients would have to be screened in order to obtain 600 randomised patients, assuming an approximate rate of 10 % screening failures.

## Results

Of the 745 patients screened, 611 patients were randomised and received the study treatment, thus constituting the safety population. Efficacy analyses were performed on the ITT population of 606 randomised patients. The PP population of 567 patients was used to perform confirmatory analyses on the primary endpoint. Patient assignment to the different populations occurred before the study blind was broken. The participant flow with the numbers of participants who were randomly assigned, received intended treatment, and were analysed for the primary outcome is represented in Additional file 1.

Demography and baseline characteristics of different treatment groups were comparable. Demographic and baseline characteristics of the ITT population are represented in Table 1. The overall mean age was 27 years (range 18–64 years), 59 % were women, and 90 % were white. The mean surgery time was 29 min, most patients (90 %) had only one mandibular molar removed, and the total number of molar extractions was one or two in 95 % of patients. Initial pain (baseline PI) was moderate or severe in 601 patients (Table 2).

**Table 1:** Demographic and baseline characteristics (ITT population)

		DKP 12.5 mg + TRAM 37.5 mg	DKP 12.5 mg + TRAM 75 mg	DKP 25 mg + TRAM 37.5 mg	DKP 25 mg + TRAM 75 mg	DKP 12.5 mg	DKP 25 mg	TRAM 37.5 mg	TRAM 75 mg	Ibuprofen	Placebo	Overall
n		60	62	63	61	60	60	59	59	60	62	606
Gender n (%)	female	34 (56.7)	38 (61.3)	36 (57.1)	34 (55.7)	36 (60.0)	43 (71.7)	38 (64.4)	27 (45.8)	40 (66.7)	33 (53.2)	359 (59.2)
	male	26 (43.3)	24 (38.7)	27 (42.9)	27 (44.3)	24 (40.0)	17 (28.3)	21 (35.6)	32 (54.2)	20 (33.3)	29 (46.8)	247 (40.8)
Ethnic origin n (%)	White	56 (93.3)	57 (91.9)	56 (88.9)	59 (96.7)	54 (90.0)	51 (85.0)	56 (94.9)	52 (88.1)	49 (81.7)	57 (91.9)	547 (90.3)
	Asian	2 (3.3)	2 (3.2)	5 (7.9)	2 (3.3)	5 (8.3)	6 (10.0)	3 (5.1)	2 (3.4)	5 (8.3)	2 (3.2)	34 (5.6)
	Black	2 (3.3)	2 (3.2)	1 (1.6)	0 (0.0)	1 (1.7)	2 (3.3)	0 (0.0)	4 (6.8)	5 (8.3)	2 (3.2)	19 (3.1)
	Other	0 (0.0)	1 (1.6)	1 (1.6)	0 (0.0)	0 (0.0)	1 (1.7)	0 (0.0)	1 (1.7)	1 (1.7)	1 (1.6)	6 (1.0)
Age (years)	mean (SD)	28.7 (7.71)	27.0 (7.66)	26.3 (7.33)	27.3 (7.55)	27.0 (9.85)	27.0 (6.94)	25.5 (7.15)	27.9 (8.04)	26.7 (6.48)	26.1 (6.64)	26.9 (7.57)
	range	18–52	18–53	18–64	18–52	18–63	18–48	18–55	18–58	18–44	18–54	18–64
BMI (kg/m^2^)	mean (SD)	23.7 (3.38)^a^	24.1 (3.69)	23.0 (2.87)	23.2 (3.19)	23.6 (3.20)	23.6 (3.30)^b^	23.0 (3.19)	24.2 (3.10)	22.4 (3.06)	22.7 (2.80)	23.3 (3.21)
	range	18–30	18–35	18–32	18–30	18–30	15–30	18–30	18–34	18–31	18–29	18–35
Surgery duration^c^	mean (SD)	29:02 (14:40)	28:45 (12:58)	29:35 (17:32)	30:57 (16:39)	29:01 (12:03)	29:39 (14:45)	27:10 (11:10)	27:37 (14:01)	29:56 (13:31)	32:28 (15:23)	29:26 (14:23)
Total third molar extractions^d^ n (%)	1	38 (63.3)	40 (64.5)	38 (60.3)	38 (62.3)	41 (68.3)	38 (63.3)	36 (61.0)	35 (59.3)	31 (51.7)	35 (56.5)	370 (61.1)
	2	17 (28.3)	20 (32.3)	23 (36.5)	18 (29.5)	17 (28.3)	20 (33.3)	22 (37.3)	21 (35.6)	26 (43.3)	22 (35.5)	206 (34.0)
	3	3 (5.0)	0 (0.0)	0 (0.0)	2 (3.3)	0 (0.0)	1 (1.7)	1 (1.7 )	2 (3.4)	2 (3.3)	3 (4.8)	14 (2.3)
	4	2 (3.3)	2 (3.2)	2 (3.2)	3 (4.9)	2 (3.3)	1 (1.7)	0 (0.0)	1 (1.7)	1 (1.7)	2 (3.2)	16 (2.6)
Lower third molar extractions n (%)	1	54 (90.0)	55 (88.7)	56 (88.9)	53 (86.9)	55 (91.7)	53 (88.3)	56 (94.9)	52 (88.1)	53 (88.3)	57 (91.9)	544 (89.8)
	2	6 (10.0)	7 (11.3)	7 (11.1)	8 (13.1)	5 (8.3)	7 (11.7)	3 (5.1)	7 (11.9)	7 (11.7)	5 (8.1)	62 (10.2)

*BMI* body mass index

^a^ *n* = 59; ^b^ *n* = 59; ^c^time is expressed in minutes and seconds; ^d^total number of third molar extractions, including also upper third molar teeth

**Table 2:** PI before randomization and before treatment administration (ITT population)

		DKP 12.5 mg + TRAM 37.5 mg	DKP 12.5 mg + TRAM 75 mg	DKP 25 mg + TRAM 37.5 mg	DKP 25 mg + TRAM 75 mg	DKP 12.5 mg	DKP 25 mg	TRAM 37.5 mg	TRAM 75 mg	Ibuprofen	Placebo	Overall
n		60	62	63	61	60	60	59	59	60	62	606
PI before randomization												
VAS	Mean (SD)	58.28 (12.50)	57.74 (11.54)	56.25 (10.79)	57.72 (11.63)	58.33 (12:49)	57.72 (12.55)	57.10 (11:73)	56.32 (11.42)	57.33 (13:46)	60.34 (13.39)	57.72 (12.13)
VRS	Moderate n (%)	48 (80.0)	53 (85.5)	48 (76.2)	48 (78.7)	45 (75.0)	47 (78.3)	56 (94.9)	47 (79.7)	51 (85.0)	46 (74.2)	489 (80.7)
	Severe n (%)	12 (20.0)	9 (14.5)	15 (23.8)	13 (21.3)	15 (25.0)	13 (21.7)	3 (5.1)	12 (20.3)	9 (15.0)	16 (25.8)	117 (19.3)
PI before treatment administration (baseline PI)												
VRS	Mild n (%)	1 (1.7)	0 (0.0)	0 (0.0)	0 (0.0)	0 (0.0)	0 (0.0)	0 (0.0)	1 (1.7)	0 (0.0)	1 (1.6)	3 (0.5)
	Moderate n (%)	40 (66.7)	43 (69.4)	39 (61.9)	41 (67.2)	35 (58.3)	43 (71.7)	40 (67.8)	35 (59.3)	39 (65.0)	33 (53.2)	388 (64.0)
	Severe n (%)	19 (31.7)	18 (29.0)	24 (38.1)	20 (32.8)	25 (41.7)	17 (28.3)	19 (32.2)	23 (39.0)	21 (35.0)	27 (43.5)	213 (35.1)
	Missing n (%)	0 (0.0)	1 (1.6)	0 (0.0)	0 (0.0)	0 (0.0)	0 (0.0)	0 (0.0)	0 (0.0)	0 (0.0)	1 (1.6)	2 (0.3)

VRS-PI measured on a 4-point VRS (0 = ‘none’ to 3 = ‘severe’). Baseline PI refers to the VRS-PI recorded immediately prior to the administration of the study medication (in contrast to VRS-PI measured before randomisation for eligibility purposes). Baseline PI was moderate or severe in 601 patients; it was reported as “mild” by 3 (0.5 %) patients and results were missing for 2 (0.3 %) patients due to compilation data error on the eDiary

Four (0.65 %) patients, out of 611 randomised, discontinued the study after randomisation, thus resulting in a total of 607 patients completing the study. One patient (allocated to DKP12.5/TRAM75) discontinued the study due to “failure of eDiary”. The other three patients (allocated respectively to DKP25/TRAM37.5, DKP25/TRAM75 and TRAM37.5) were “lost to follow-up”. Three of these four patients attended Visit 3.

### Efficacy results

#### Primary endpoint

**Fig. 1:**
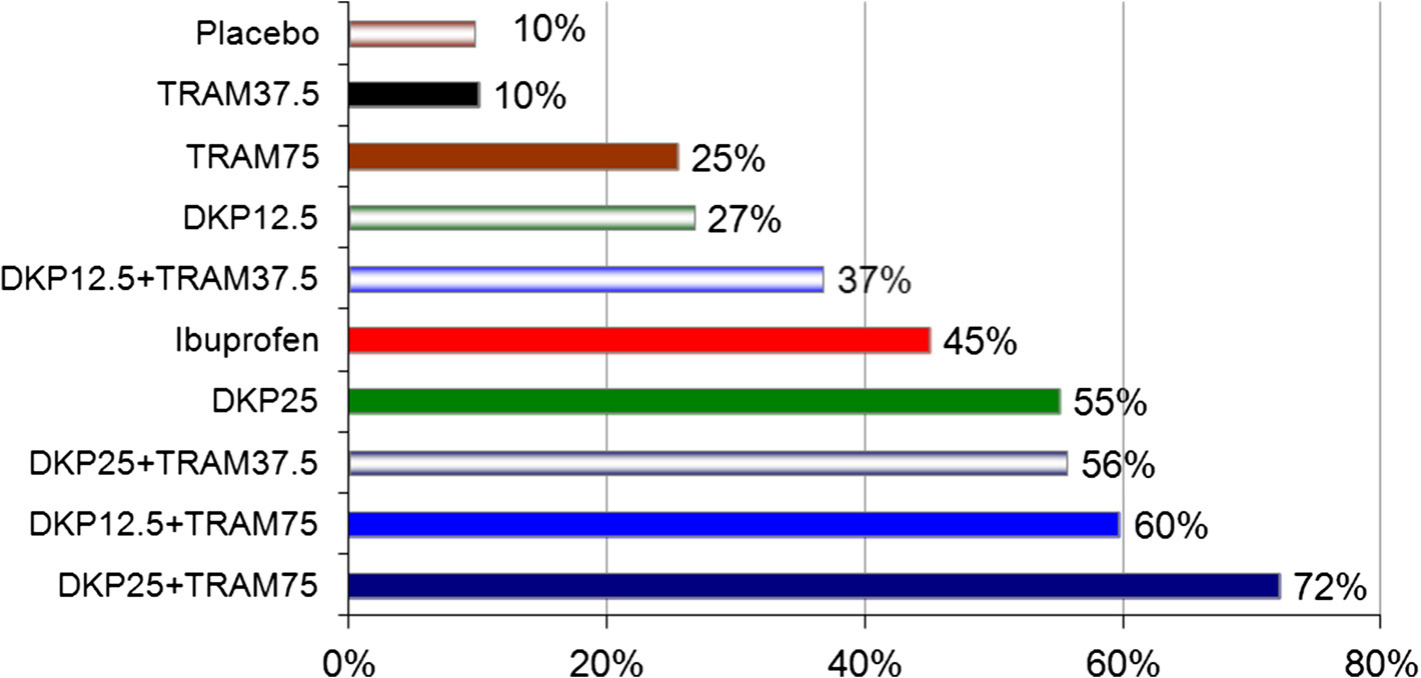
Percentage of patients showing response (≥50 % max TOTPAR) over 6 h post-dose (Primary Endpoint). Maximum TOTPAR corresponds to the theoretical maximum possible time-weighted sum of the PAR scores, measured on a 5-point VRS (0 = ‘none’ to 4 = ‘complete’)

The percentage of patients with ≥ 50 % max TOTPAR over six hours post-dose was significantly superior to placebo for all DKP/TRAM combinations and also for DKP25 (*p* < 0.0001 for each comparison, except *p* = 0.0009 for DKP12.5/TRAM37.5), with the highest percentage of responders achieved in the DKP25/TRAM75 group (72 % versus 10 % in the placebo group; Fig. 1 and Additional file 2.

**Fig. 2:**
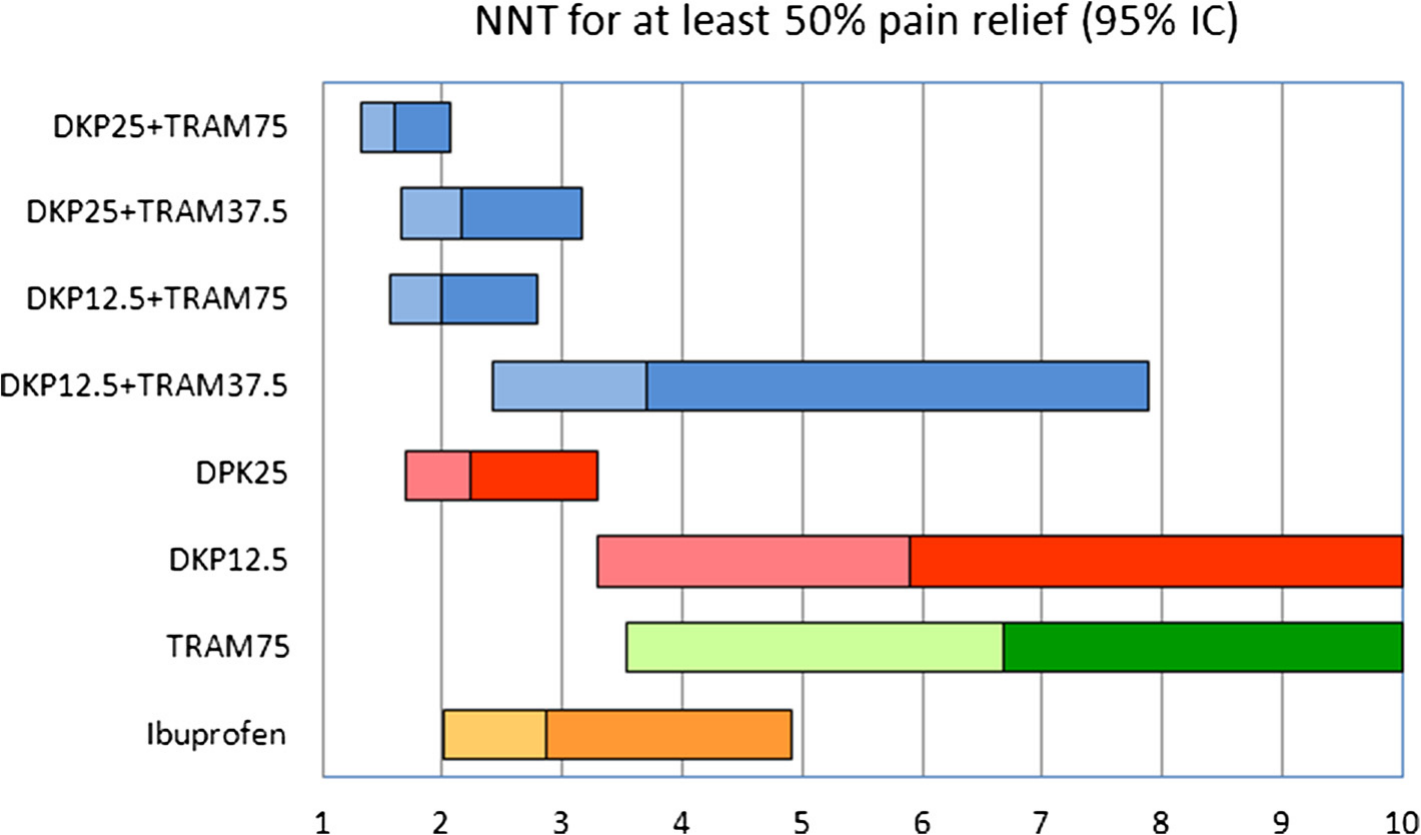
NNT for ≥ 50 % max TOTPAR compared with placebo over six hours post dose. Maximum TOTPAR corresponds to the theoretical maximum possible time-weighted sum of the PAR scores, measured on a 5-point VRS (0 = ‘none’ to 4 = ‘complete’). Bars show 95 % confidence interval of NNT, with colour change as point estimate (Note that TRAM37.5 was not significantly better than placebo)

Detailed ER, RR and NNT results at six hours are presented in Additional file 3. DKP25/TRAM75 also had the highest RR [7.2 (95 % CI: 3.3 to 15.7)] and the lowest NNT [1.6 (95 % CI: 1.3 to 2.1)]. No other point estimate for NNT was below 2.0 (Fig. 2). The NNT for DKP25/TRAM75 was significantly better than other NNT values (*p* < 0.05, z-test), except for DKP25/TRAM37.5, DKP12.5/TRAM75 and DKP25.

The percentage of responders (≥50 % max TOTPAR) was significantly superior to placebo for all DKP/TRAM combinations and for both doses of DKP in monotherapy (*p* < 0.0001) over four hours and it remained significantly superior for DKP25/TRAM75, DKP12.5/TRAM75, DKP25/TRAM37.5 (*p* < 0.0001) and DKP25 (*p* = 0.0012) over eight hours; and for DKP25/TRAM75 (*p* = 0.0002), DKP12.5/TRAM75 (*p* = 0.0004) and DKP25/TRAM37.5 (*p* = 0.0028) over 12 h. The highest percentage of responders over four, eight and 12 h was achieved with DKP25/TRAM75 (79 %, 54 % and 38 % respectively) versus 6.5 % with placebo. Results are represented in Additional file 4 and Additional file 5**.**

The active control, ibuprofen 400 mg, was statistically superior to placebo (*p* < 0.0001), thus validating the pain model. Analyses run on the PP population confirmed the primary efficacy results.

#### Secondary endpoints

**Fig. 3:**
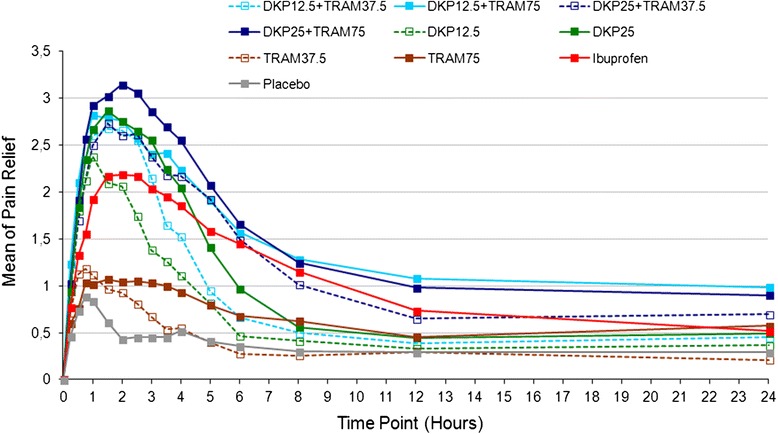
Time course of mean PAR scores (0–24 h). PAR measured on a 5-point VRS (0 = ‘none’ to 4 = ‘complete’)

The time course of mean PAR and PI over the whole 24 h post-dose demonstrated rapid onset of pain relief with dexketoprofen alone or in combination, and that the addition of tramadol to dexketoprofen resulted both in greater peak pain relief and greater pain relief over the longer term, particularly at times longer than six hours post dose (Fig. 3, and Additional file 6).

The analysis of summary efficacy measures (SPID, % max SPID, TOTPAR and % max TOTPAR; Additional file 7, Additional file 8, Additional file 9, Additional file 10 and Additional file 11) showed that all DKP/TRAM combinations and both doses of DKP in monotherapy were significantly superior to placebo (*p* < 0.01) except for DKP12.5 over 12 h, with the best results achieved with DKP25/TRAM75. The time course of mean SPID and mean TOTPAR are represented in Additional file 12 and Additional file 13.

**Fig. 4:**
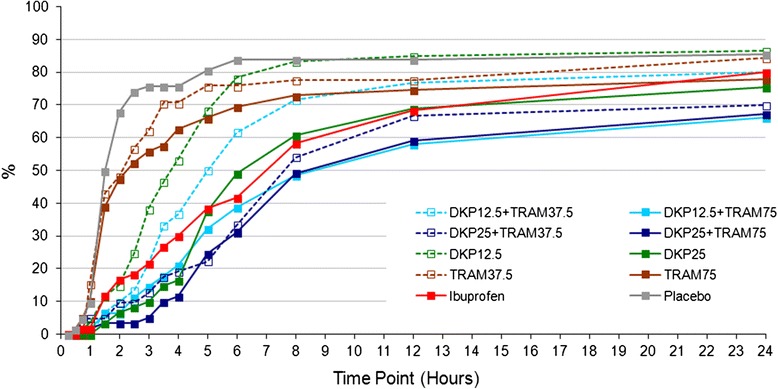
Cumulative frequency (Kaplan-Meier estimation) of RM intake (0–24 h)

The time to RM was significantly longer (*p* < 0.005) for all active treatments (except for both doses of TRAM.HCl in monotherapy) than for placebo, with DKP12.5/TRAM75 and DKP25/TRAM75 presenting the longest value (median time, [95 % CI]: 8.5 h [5.9 to 13.0] and 8.1 h [6.3 to 13.4] respectively, versus 1.4 h [1.2 to 1.8] in the placebo group). Fig. 4 shows the proportion of patients remedicating over time in each group, with additional information in Additional file 14 and Additional file 15.

The percentage of patients requiring RM (Additional file 16 and Additional file 17) over four and six hours was significantly smaller (*p* < 0.00639) for DKP12.5/TRAM75, DKP25/TRAM37.5, and DKP25/TRAM75 (also for DKP25 over four hours) than for placebo (over six hours 47 %, 40 %, and 38 % for each combination respectively versus 73 % for placebo). The difference remained significant for DKP25/TRAM75 over eight hours (48 % versus 73 %) and for DKP25/TRAM37.5 over 12 and 24 h (49 % versus 74 % for both time points).

**Fig. 5:**
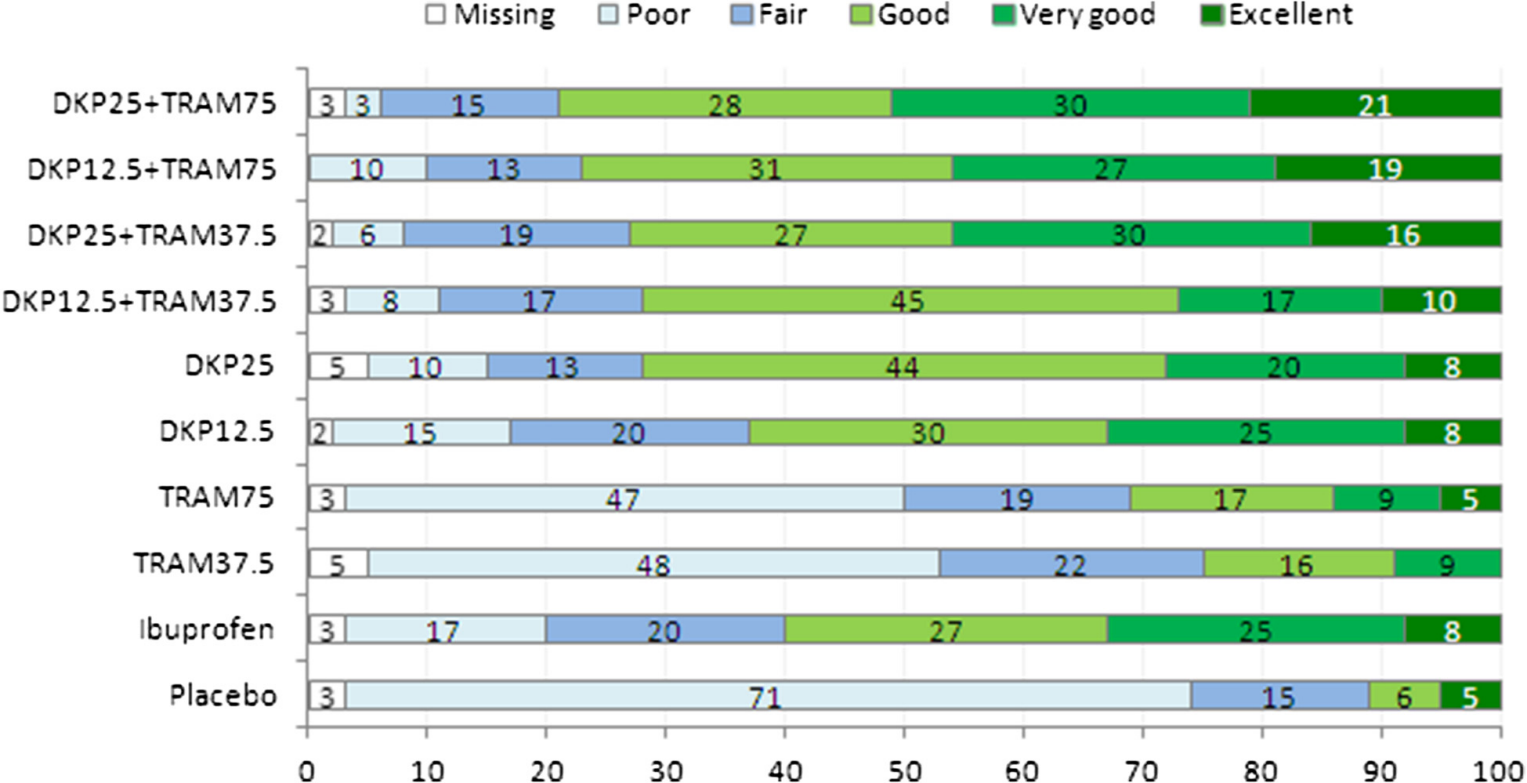
PGE at the end of the assessment period. PGE measured on a 5-point VRS (1 = ‘poor’ to 5 = ‘excellent’)

The PGE of the study medication at the end of the assessment period is represented in Fig. 5. All treatments (except TRAM37.5) were significantly superior to placebo (*p* < 0.01), with the highest scores in the DKP25/TRAM75 group. The percentage of patients with ‘good’ to ‘excellent’ PGE response was 79 % in the DKP25/TRAM75 group versus 11 % in the placebo group. The percentage of patients with ‘very good’ and ‘excellent’ PGE was 51 % in the DKP25/TRAM75 group versus 4.8 % in the placebo group.

The analysis of dose–response relationship between the tested study drugs and the active control showed that DKP25/TRAM75 was the only combination that was significantly superior to ibuprofen 400 mg (*p* = 0.0028).

### Safety results

Of 611 patients treated, 40 (6.5 %) reported a total of 63 adverse reactions (ADRs), the most frequent being vomiting (21 patients; 3.4 %), nausea (14 patients; 2.3 %), dizziness (11 patients; 1.8 %) and somnolence (5 patients; 0.8 %) (Table 3). Apart from one case of “severe” somnolence in the DKP12.5/TRAM75 group (1.6 %), all ADRs were considered “mild” (45 ADRs, 71 %) or “moderate” (17 ADRs, 27 %) in intensity.

**Table 3:** ADRs - by system organ class /preferred term, by treatment group and overall

System organ class	Preferred term	DKP 12.5 mg + TRAM 37.5 mg	DKP 12.5 mg + TRAM 75 mg	DKP 25 mg + TRAM 37.5 mg	DKP 25 mg + TRAM 75 mg	DKP 12.5 mg	DKP 25 mg	TRAM 37.5 mg	TRAM 75 mg	Ibuprofen	Placebo	Overall
		*n* = 61	*n* = 63	*n* = 63	*n* = 61	*n* = 60	*n* = 61	*n* = 59	*n* = 60	*n* = 61	*n* = 62	*n* = 611
Gastrointestinal disorders		3 (4.9)|5	4 (6.3)|4	3 (4.8)|3	6 (9.8)|8	1 (1.7)|1	3 (4.9)|3	0	8 (13.3)|13	1 (1.6)|1	0	29 (4.7)|38
	Abdominal pain upper	0	0	0	0	0	0	0	1 (1.7)|1	0	0	1 (0.2)|1
	Nausea	3 (4.9)|3	0	0	3 (4.9)|3	1 (1.7)|1	1 (1.6)|1	0	6 (10.0)|7	0	0	14 (2.3)|15
	Vomiting	2 (3.3)|2	4 (6.3)|4	3 (4.8)|3	4 (6.6)|5	0	2 (3.3)|2	0	5 (8.3)|5	1 (1.6)|1	0	21 (3.4)|22
General disorders and administration site conditions		0	1 (1.6)|1	0	2 (3.3)|2	0	1 (1.6)|1	1 (1.7)|1	0	1 (1.6)|1	0	6 (1.0)|6
	Chills	0	1 (1.6)|1	0	0	0	0	0	0	0	0	1 (0.2)|1
	Discomfort	0	0	0	1 (1.6)|1	0	0	0	0	1 (1.6)|1	0	2 (0.3)|2
	Feeling abnormal	0	0	0	1 (1.6)|1	0	0	0	0	0	0	1 (0.2)|1
	Pyrexia	0	0	0	0	0	1 (1.6)|1	1 (1.7)|1	0	0	0	2 (0.3)|2
Nervous System disorders		1 (1.6)|1	4 (6.3)|4	1 (1.6)|1	3 (4.9)|3	0	0	2 (3.4)|2	3 (5.0)|4	2 (3.3)|2	0	16 (2.6)|17
	Dizziness	1 (1.6)|1	2 (3.2)|2	0	2 (3.3)|2	0	0	1 (1.7)|1	3 (5.0)|3	2 (3.3)|2	0	11 (1.8)|11
	Headache	0	0	0	0	0	0	1 (1.7)|1	0	0	0	1 (0.2)|1
	Somnolence	0	2 (3.2)|2	1 (1.6)|1	1 (1.6)|1	0	0	0	1 (1.7)|1	0	0	5 (0.8)|5
Psychiatric disorders		0	0	0	0	0	0	0	1 (1.7)|1	0	0	1 (0.2)|1
	Nervousness	0	0	0	0	0	0	0	1 (1.7)|1	0	0	1 (0.2)|1
Vascular disorders		0	0	0	0	0	0	0	1 (1.7)|1	0	0	1 (0.2)|1
	Hypotension	0	0	0	0	0	0	0	1 (1.7)|1	0	0	1 (0.2)|1
Overall		3 (4.9)|6	6 (9.5)|9	4 (6.3)|4	7 (11.5)|13	1 (1.7)|1	3 (4.9)|4	3 (5.1)|3	10 (16.7)|19	3 (4.9)|4	0	40 (6.5)|63

Results are expressed as number of patients (% of exposed) | number of events

Only one serious adverse event (SAE) was reported in one patient (allocated to the TRAM75 group), consisting in dizziness of mild intensity, which required hospitalization for monitoring and resolved spontaneously. The event was assessed as “possibly related” to the study medication. Dizziness is a commonly reported ADR associated to the use of tramadol, occurring in more than 10 % of patients, according to the authorized Summary of Product Characteristics.

The highest incidence of ADRs was reported in the TRAM75 group (17 %). This incidence was higher than reported in groups receiving tramadol 75 mg in combination (i.e. DKP12.5/TRAM75 and DKP25/TRAM75) for which the incidence was of 9.5 % and 12 % of patients, respectively.

No deaths or other significant AEs occurred. No patient discontinued because of AEs. There were no clinically relevant changes in the VS, physical examination, 12-lead ECG or laboratory safety tests versus baseline. Overall, all treatments were safe and well tolerated, with all DKP/TRAM combinations presenting a safety and tolerability profile fully in line with that previously established for the single agents.

## Discussion

The results of this trial demonstrate good results from a combination of a rapidly-acting NSAID, dexketoprofen, with a longer lasting opioid with additional enhancement of serotonin and norepinephrine transmission [27]. The particular combination of dexketoprofen trometamol 25 mg plus tramadol hydrochloride 75 mg delivered rapid initial pain relief, low (good) NNT for at least 50 % max TOTPAR over six hours, long duration, and a small proportion of patients remedicating. The NNT of 1.6 and the eight hours before 50 % of patients remedicated were comparable to or better than most other oral treatments for acute postoperative pain [3].

This makes DKP25/TRAM75 a good candidate for a combination treatment in acute pain. In part, this is because the combination unites two commonly used drugs (dexketoprofen is the active chiral form of ketoprofen) with known properties. This is important because new rare but serious adverse events are less likely than, say, with a new chemical. But efficacy, tolerability, and safety need to be tested in more trials and more patients, perhaps especially in multiple dose studies that capture more information than a single dose efficacy study can do.

Although this trial was performed to high quality standards, and was relatively large with over 600 patients recruited, the modest size of each group means that extrapolation to clinical practice should not be undertaken. However, the NNT for ibuprofen 400 mg was measured in this trial as 2.9 (95 % confidence interval 2.0 to 4.9); this is comfortably close to that of 2.5 (2.4 to 2.6) found in a meta-analysis of over 6000 patients [20], and that for dexketoprofen trometamol 25 mg of 2.2 (1.7 to 3.3) close to that of 3.2 (2.6 to 4.1) in another meta-analysis [13], or 2.6 (2.0 to 3.5) in dental pain in an analysis from clinical trial reports [14]. Information on tramadol hydrochloride 75 mg in single dose studies is limited and variable [16, 17], but the wide confidence interval around the measured NNT of 6.7 (3.5 to 60) encompasses existing estimates.

The other interesting, and perhaps important, result of this trial was that the pre-trial estimates of efficacy of the combinations, determined using a formula derived from empirical data, proved to be reasonably accurate predictors of measured efficacy. Because of limited efficacy data for single dose dexketoprofen and tramadol at the doses envisaged, the pre-trial estimates could be little more than informed guesses, but they were not inaccurate.

## Conclusion

Dexketoprofen trometamol 25 mg combined with tramadol hydrochloride 75 mg provided good all-round analgesia, with rapid onset and long duration in a model of moderate to severe pain. The results of the dose finding study are consistent with pre-trial calculations based on empirical formulae.

## Additional files

Additional file 1:
**Study CONSORT flow diagram.** Participant flow with the numbers of participants who were randomly assigned, received intended treatment, and were analysed for the primary outcome. Additional file 2:
**Cumulative percentage of patients showing response (≥50 % max TOTPAR) over 6 h post-dose (Primary Endpoint).** Maximum TOTPAR corresponds to the theoretical maximum possible time-weighted sum of the PAR scores, measured on a 5-point VRS (0 = ‘none’ to 4 = ‘complete’). Additional file 3:
**Effect size on the percentage of patients showing response (≥50 % max TOTPAR) over 6 h - Estimated ER, RR and NNT (95 % CI).**
Additional file 4:
**Percentage of patients showing response (≥50 % max TOTPAR) over 4 h, 6 h (Primary Endpoint), 8 h and 12 h post-dose.**
Additional file 5:
**Statistical Analysis of the percentage of patients showing response (≥50 % max TOTPAR) over 4 h, 6 h (Primary Endpoint), 8 h and 12 h post-dose.**
Additional file 6:
**Time course of mean VRS-PI scores (0–24 h).** VRS-PI measured on a 4-point VRS (0 = ‘none’ to 3 = ‘severe’). Additional file 7:
**Descriptive statistics (mean and SD) for SPID, % max SPID, TOTPAR and % max TOTPAR over 4, 6, 8 and 12 h.**
Additional file 8:
**Statistical Analysis of TOTPAR over 4, 6, 8 and 12 h.**
Additional file 9:
**Statistical analysis of percentage of max TOTPAR over 4, 6, 8 and 12 h.**
Additional file 10:
**Statistical analysis of SPID over 4, 6, 8 and 12 h.**
Additional file 11:
**Statistical analysis of percentage of max SPID over 4, 6, 8 and 12 h.**
Additional file 12:
**Time course of mean SPID (0–12 h).** PI measured on a 4-point VRS (0 = ‘none’ to 3 = ‘severe’); PID = PI t_0h_ (baseline PI) – PI t (PI at time point t). (TIFF 86 kb) Additional file 13:
**Time course of mean TOTPAR (0–12 h).** PAR measured on a 5-point VRS (0 = ‘none’ to 4 = ‘complete’). Additional file 14:
**Kaplan-Meier survival distribution of the time to RM (0–24 h).** Time to RM is defined as the time elapsed between the treatment administration and the first RM use; the survival distribution estimates the probability of ‘no use of RM’ at each time point. Additional file 15:
**Time to RM (Kaplan-Meier estimation) over 24 h.**
Additional file 16:
**Cumulative percentage of patients who required RM over 4, 6, 8, 12 and 24 h.**
Additional file 17:
**Statistical analysis of percentage of patients who required RM over 4, 6, 8, 12 and 24 h.**
