# Supplementary material for: Dexketoprofen/tramadol: randomised double-blind trial and confirmation of empirical theory of combination analgesics in acute pain
Source: J Headache Pain. 2015 Jun 27;16:60. doi: 10.1186/s10194-015-0541-5 (PMC4485659; doi:10.1186/s10194-015-0541-5)
Supplement: Additional file 1: — Study CONSORT flow diagram. Participant flow with the numbers of participants who were randomly assigned, received intended treatment, and were analysed for the primary outcome. [file 10194_2015_541_MOESM1_ESM.docx]

**a)** One patient received the incorrect kit study treatment: the patient was randomised to receive DKP25 but received TRAM37.5 instead; **Safety population:** All patients randomised who received the study treatment; **Intention-to-Treat (ITT) population:** All patients of the safety population with at least one post-dose assessment; **Per Protocol (PP) population:** All patients of the ITT population with no major protocol deviations; **f.u.:** follow-up.

**Enrolment**

**Allocation**

**Analysis Populations**

**Follow-up**
