# Supplementary figures and images for: Dexketoprofen/tramadol: randomised double-blind trial and confirmation of empirical theory of combination analgesics in acute pain
Source: J Headache Pain. 2015 Jun 27;16:60. doi: 10.1186/s10194-015-0541-5 (PMC4485659; doi:10.1186/s10194-015-0541-5)

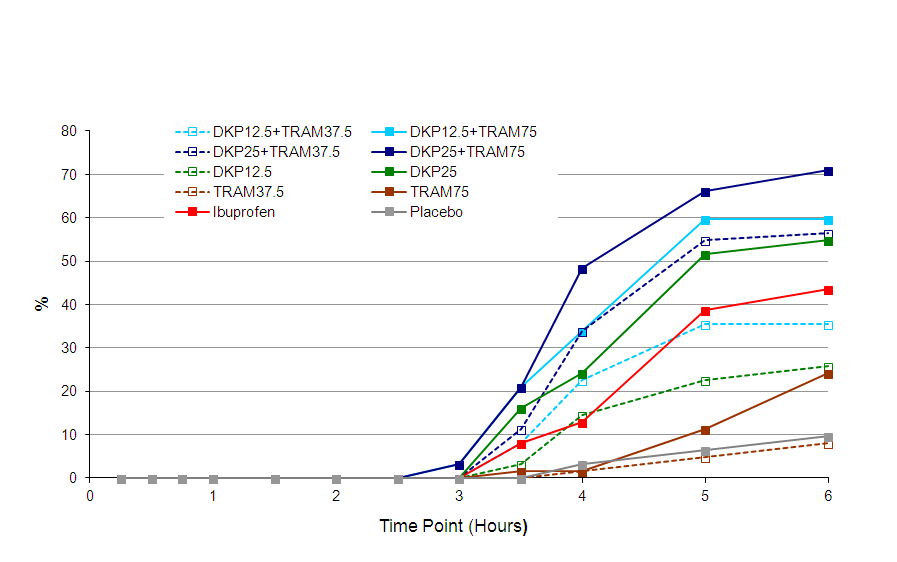

Supplement: Additional file 2: — Cumulative percentage of patients showing response (≥50 % max TOTPAR) over 6 h post-dose (Primary Endpoint). Maximum TOTPAR corresponds to the theoretical maximum possible time-weighted sum of the PAR scores, measured on a 5-point VRS (0 = ‘none’ to 4 = ‘complete’). [file 10194_2015_541_MOESM2_ESM.tiff]

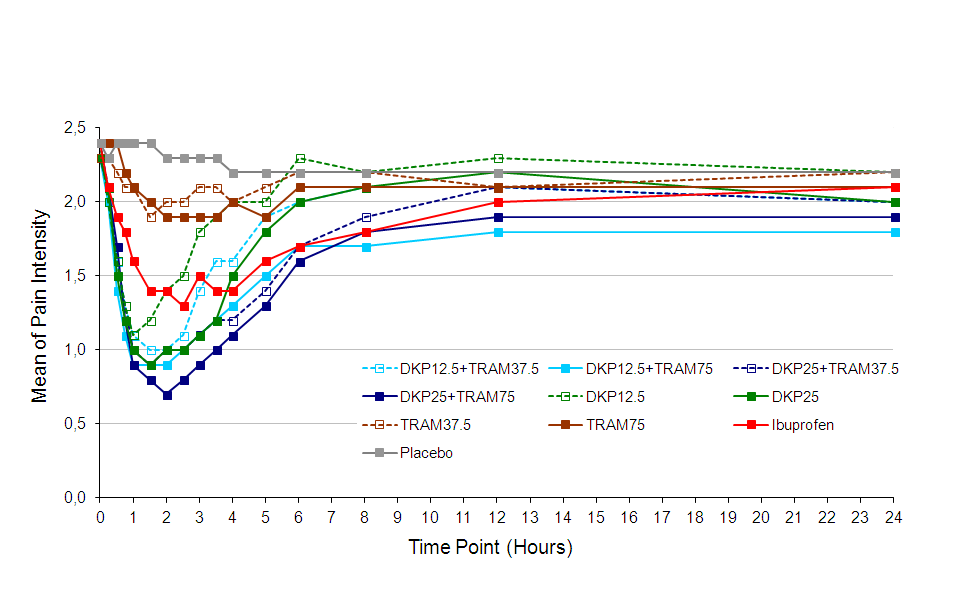

Supplement: Additional file 6: — Time course of mean VRS-PI scores (0–24 h). VRS-PI measured on a 4-point VRS (0 = ‘none’ to 3 = ‘severe’). [file 10194_2015_541_MOESM6_ESM.tiff]

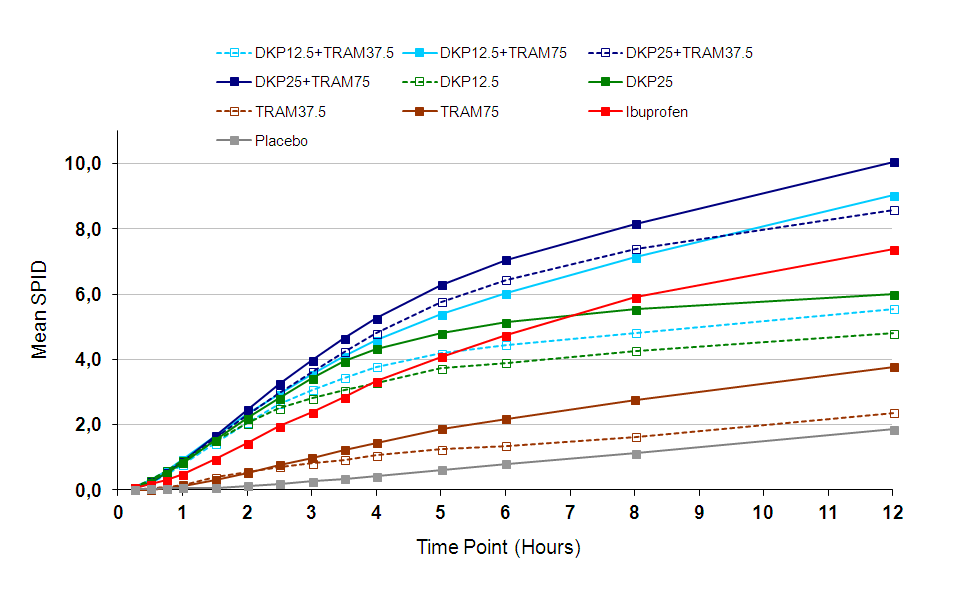

Supplement: Additional file 12: — Time course of mean SPID (0–12 h). PI measured on a 4-point VRS (0 = ‘none’ to 3 = ‘severe’); PID = PI t0h (baseline PI) – PI t (PI at time point t). (TIFF 86 kb) [file 10194_2015_541_MOESM12_ESM.tiff]

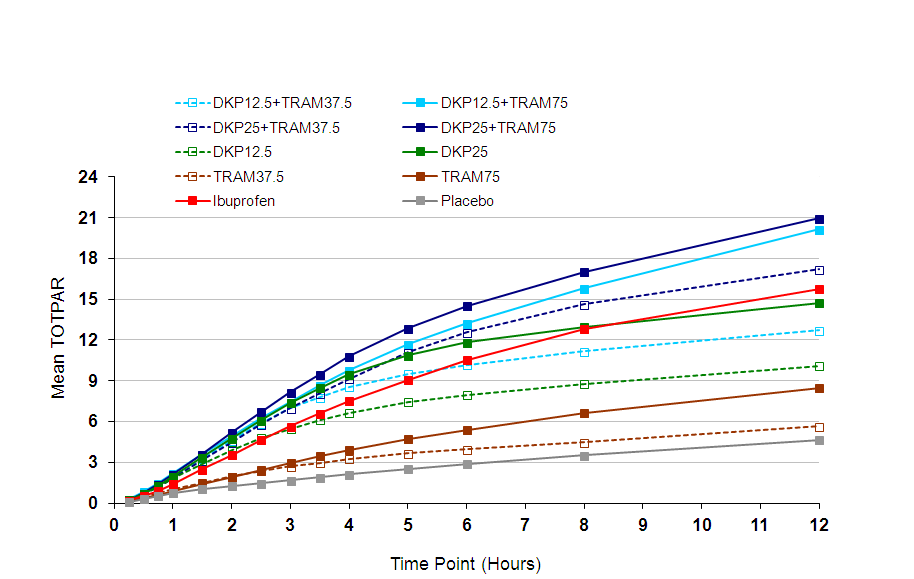

Supplement: Additional file 13: — Time course of mean TOTPAR (0–12 h). PAR measured on a 5-point VRS (0 = ‘none’ to 4 = ‘complete’). [file 10194_2015_541_MOESM13_ESM.tiff]

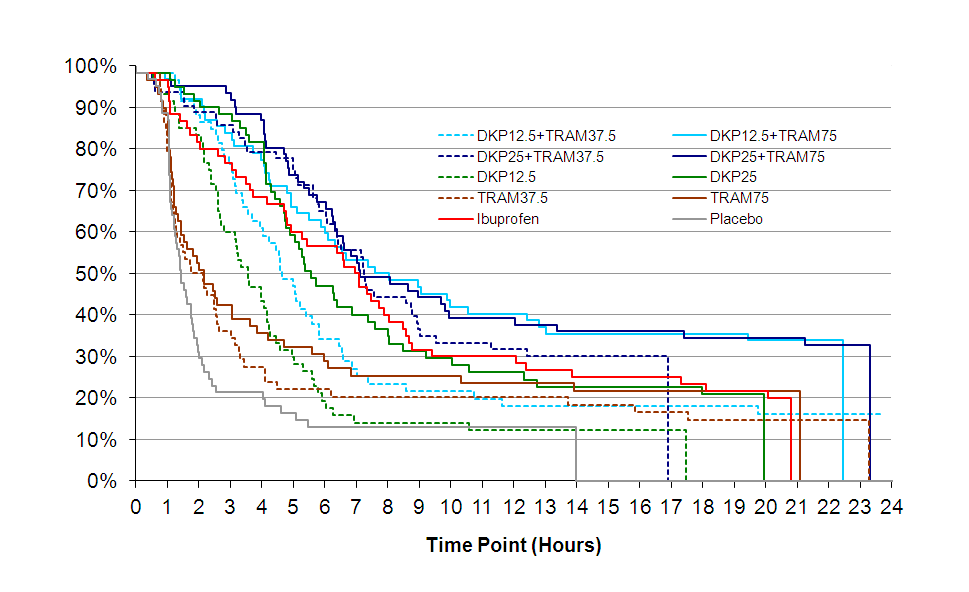

Supplement: Additional file 14: — Kaplan-Meier survival distribution of the time to RM (0–24 h). Time to RM is defined as the time elapsed between the treatment administration and the first RM use; the survival distribution estimates the probability of ‘no use of RM’ at each time point. [file 10194_2015_541_MOESM14_ESM.tiff]
