# Supplementary material for: Dexketoprofen/tramadol: randomised double-blind trial and confirmation of empirical theory of combination analgesics in acute pain
Source: J Headache Pain. 2015 Jun 27;16:60. doi: 10.1186/s10194-015-0541-5 (PMC4485659; doi:10.1186/s10194-015-0541-5)
Supplement: Additional file 3: — Effect size on the percentage of patients showing response (≥50 % max TOTPAR) over 6 h - Estimated ER, RR and NNT (95 % CI). [file 10194_2015_541_MOESM3_ESM.docx]

Additional file 3: Effect Size on the percentage of patients showing response (≥ 50% max TOTPAR) over 6 hours - Estimated ER, RR and NNT (95% CI).

|  | | | DKP 12.5mg + TRAM 37.5mg | DKP 12.5mg + TRAM 75mg | DKP 25mg + TRAM 37.5mg | DKP 25mg + TRAM 75mg | DKP 12.5mg | DKP 25mg | TRAM 37.5mg | **TRAM 75mg** | Ibuprofen |
| --- | --- | --- | --- | --- | --- | --- | --- | --- | --- | --- | --- |
| Estimated ER (95% CI) | | | | | | | | | | | |
| **6 hours** | | control | Placebo | Placebo | Placebo | Placebo | Placebo | Placebo | Placebo | Placebo | Placebo |
|  |  | estimate | 0.37 | 0.60 | 0.56 | 0.72 | 0.27 | 0.55 | 0.10 | 0.25 | 0.45 |
|  |  | CI lower limit | 0.25 | 0.48 | 0.44 | 0.61 | 0.16 | 0.42 | 0.02 | 0.14 | 0.32 |
|  |  | CI upper limit | 0.49 | 0.72 | 0.68 | 0.83 | 0.38 | 0.68 | 0.18 | 0.36 | 0.58 |
| Estimated RR (95% CI) | | | | | | | | | | | |
| **6 hours** | | control | Placebo | Placebo | Placebo | Placebo | Placebo | Placebo | Placebo | Placebo | Placebo |
|  |  | estimate | 3.70 | 6.00 | 5.60 | 7.20 | 2.70 | 5.50 | 1.00 | 2.50 | 4.50 |
|  |  | CI lower limit | 1.61 | 2.73 | 2.54 | 3.31 | 1.13 | 2.49 | 0.34 | 1.04 | 2.00 |
|  |  | CI upper limit | 8.49 | 13.19 | 12.36 | 15.65 | 6.44 | 12.17 | 2.93 | 6.01 | 10.12 |
| Estimated NNT (95% CI) | | | | | | | | | | | |
| **6 hours** | **control** | | Placebo | Placebo | Placebo | Placebo | Placebo | Placebo | Placebo | Placebo | Placebo |
|  | estimate | | 3.70 | 2.00 | 2.17 | 1.61 | 5.88 | 2.22 | - | 6.67 | 2.86 |
|  | CI lower limit | | 2.42 | 1.55 | 1.66 | 1.32 | 3.28 | 1.68 | NA | 3.53 | 2.01 |
|  | CI upper limit | | 7.88 | 2.80 | 3.16 | 2.06 | 28.48 | 3.29 | NA | 60.10 | 4.91 |

NA: Not Applicable; ER: event rate; RR: relative risk; NNT: number needed to treat; CI: confidence interval. Maximum TOTPAR corresponds to the theoretical maximum possible time-weighted sum of the PAR scores, measured on a 5-point VRS (0=‘none’ to 4=‘complete’). RR values greater than 1 show that the experimental drug is better than placebo. Statistical significance is assumed if the 95% CI does not include 1; NNT indicates the number of patients who need to receive the treatment for 1 to achieve the outcome, in comparison with placebo; a missing NNT means that the effect using the active treatment is less or equal to the effect of using placebo.
