# Supplementary material for: Dexketoprofen/tramadol: randomised double-blind trial and confirmation of empirical theory of combination analgesics in acute pain
Source: J Headache Pain. 2015 Jun 27;16:60. doi: 10.1186/s10194-015-0541-5 (PMC4485659; doi:10.1186/s10194-015-0541-5)
Supplement: Additional file 4: — Percentage of patients showing response (≥50 % max TOTPAR) over 4 h, 6 h (Primary Endpoint), 8 h and 12 h post-dose. [file 10194_2015_541_MOESM4_ESM.docx]

Additional file 4: Percentage of patients showing response (≥ 50% max TOTPAR) over 4 hours, 6 hours (Primary Endpoint), 8 hours and 12 hours post-dose.

| **Responders**  **(≥ 50% maxTOTPAR)**  n (%) | DKP 12.5mg + TRAM 37.5mg | DKP 12.5mg + TRAM 75mg | DKP 25mg + TRAM 37.5mg | DKP 25mg + TRAM 75mg | DKP 12.5mg | DKP 25mg | TRAM 37.5mg | **TRAM 75mg** | Ibuprofen | Placebo | Overall |
| --- | --- | --- | --- | --- | --- | --- | --- | --- | --- | --- | --- |
|  | n=60 | n=62 | n=63 | n=61 | n=60 | n=60 | n=59 | n=59 | n=60 | n=62 | n=606 |
| **4 hours** | 38  (63.3) | 45  (72.6) | 41  (65.1) | 48  (78.7) | 24  (40.0) | 39  (65.0) | 7  (11.9) | 14  (23.7) | 34  (56.7) | 4  (6.5) | 294 (48.5) |
| **6 hours**  **(Primary Endpoint)** | 22  (36.7) | 37  (59.7) | 35  (55.6) | 44  (72.1) | 16  (26.7) | 33  (55.0) | 6  (10.2) | 15  (25.4) | 27  (45.0) | 6  (9.7) | 241  (39.8) |
| **8 hours** | 13  (21.7) | 30  (48.4) | 28  (44.4) | 33  (54.1) | 10  (16.7) | 19  (31.7) | 4  (6.8) | 12  (20.3) | 20  (33.3) | 4  (6.5) | 173 (28.5) |
| **12 hours** | 7  (11.7) | 22  (35.5) | 18  (28.6) | 23  (37.7) | 6  (10.0) | 8  (13.3) | 3  (5.1) | 9  (15.3) | 15  (25.0) | 4  (6.5) | 115 (19.0) |

Maximum TOTPAR corresponds to the theoretical maximum possible time-weighted sum of the PAR scores, measured on a 5-point VRS (0=‘none’ to 4=‘complete’).
