# Supplementary material for: Dexketoprofen/tramadol: randomised double-blind trial and confirmation of empirical theory of combination analgesics in acute pain
Source: J Headache Pain. 2015 Jun 27;16:60. doi: 10.1186/s10194-015-0541-5 (PMC4485659; doi:10.1186/s10194-015-0541-5)
Supplement: Additional file 5: — Statistical Analysis of the percentage of patients showing response (≥50 % max TOTPAR) over 4 h, 6 h (Primary Endpoint), 8 h and 12 h post-dose. [file 10194_2015_541_MOESM5_ESM.docx]

Additional file 5. Statistical Analysis of the percentage of patients showing response (≥ 50% max TOTPAR) over 4 hours, 6 hours (Primary Endpoint), 8 hours and 12 hours post-dose.

| **Percentage of responders** | **Treatment** | **Control** | **Estimate** | **Standard Error** | **CI Lower Limit** | **CI Upper Limit** | **Pr > \|z\|** | **Significant** |
| --- | --- | --- | --- | --- | --- | --- | --- | --- |
| **4 h.** | **DKP12.5+Tram37.5** | **Placebo** | 3.22 | 0.58 | 1.63 | 4.81 | < 0.0001 | Yes |
|  | **DKP12.5+Tram75** | **Placebo** | 3.65 | 0.59 | 2.04 | 5.26 | < 0.0001 | Yes |
|  | **DKP25+Tram37.5** | **Placebo** | 3.30 | 0.58 | 1.71 | 4.88 | < 0.0001 | Yes |
|  | **DKP25+Tram75** | **Placebo** | 3.98 | 0.60 | 2.33 | 5.63 | < 0.0001 | Yes |
|  | **DKP12.5** | **Placebo** | 2.27 | 0.58 | 0.69 | 3.85 | < 0.0001 | Yes |
|  | **DKP25** | **Placebo** | 3.29 | 0.58 | 1.70 | 4.88 | < 0.0001 | Yes |
|  | **Tram37.5** | **Placebo** | 0.67 | 0.66 | -1.12 | 2.46 | 0.30738 | No |
|  | **Tram75** | **Placebo** | 1.51 | 0.60 | -0.13 | 3.14 | 0.01215 | No |
| **6 h**  **(primary endpoint)** | **DKP12.5+Tram37.5** | **Placebo** | 1.69 | 0.51 | 0.31 | 3.07 | 0.0009 | Yes |
|  | **DKP12.5+Tram75** | **Placebo** | 2.63 | 0.50 | 1.26 | 3.99 | < 0.0001 | Yes |
|  | **DKP25+Tram37.5** | **Placebo** | 2.46 | 0.50 | 1.10 | 3.82 | < 0.0001 | Yes |
|  | **DKP25+Tram75** | **Placebo** | 3.18 | 0.52 | 1.78 | 4.59 | < 0.0001 | Yes |
|  | **DKP12.5** | **Placebo** | 1.22 | 0.52 | -0.19 | 2.64 | 0.0186 | No |
|  | **DKP25** | **Placebo** | 2.43 | 0.50 | 10.70 | 3.8 | < 0.0001 | Yes |
|  | **Tram37.5** | **Placebo** | 0.06 | 0.61 | -1.60 | 1.71 | 0.9279 | No |
|  | **Tram75** | **Placebo** | 1.16 | 0.52 | -0.27 | 2.58 | 0.0270 | No |
| **8 h.** | **DKP12.5+Tram37.5** | **Placebo** | 1.39 | 0.60 | -0.26 | 3.04 | 0.02158 | No |
|  | **DKP12.5+Tram75** | **Placebo** | 2.61 | 0.58 | 1.04 | 4.18 | < 0.0001 | Yes |
|  | **DKP25+Tram37.5** | **Placebo** | 2.45 | 0.58 | 0.88 | 4.02 | < 0.0001 | Yes |
|  | **DKP25+Tram75** | **Placebo** | 2.84 | 0.58 | 1.26 | 4.41 | < 0.0001 | Yes |
|  | **DKP12.5** | **Placebo** | 1.06 | 0.62 | -0.63 | 2.76 | 0.08709 | No |
|  | **DKP25** | **Placebo** | 1.91 | 0.59 | 0.30 | 3.51 | 0.00117 | Yes |
|  | **Tram37.5** | **Placebo** | 0.05 | 0.73 | -1.94 | 2.05 | 0.94214 | No |
|  | **Tram75** | **Placebo** | 1.31 | 0.61 | -0.35 | 2.97 | 0.03184 | No |
| **12 h.** | **DKP12.5+Tram37.5** | **Placebo** | 0.65 | 0.65 | -1.14 | 2.44 | 0.32116 | No |
|  | **DKP12.5+Tram75** | **Placebo** | 2.08 | 0.58 | 0.49 | 3.66 | 0.00035 | Yes |
|  | **DKP25+Tram37.5** | **Placebo** | 1.76 | 0.59 | 0.16 | 3.36 | 0.00277 | Yes |
|  | **DKP25+Tram75** | **Placebo** | 2.17 | 0.58 | 0.59 | 3.76 | 0.00018 | Yes |
|  | **DKP12.5** | **Placebo** | 0.48 | 0.67 | -1.36 | 2.31 | 0.4783 | No |
|  | **DKP25** | **Placebo** | 0.80 | 0.64 | -0.95 | 2.55 | 0.211 | No |
|  | **Tram37.5** | **Placebo** | -0.25 | 0.79 | -2.40 | 1.89 | 0.74806 | No |
|  | **Tram75** | **Placebo** | 0.96 | 0.63 | -0.76 | 2.68 | 0.12851 | No |

Maximum TOTPAR corresponds to the theoretical maximum possible time-weighted sum of the PAR scores, measured on a 5-point VRS (0=‘none’ to 4=‘complete’).
