# Supplementary material for: Dexketoprofen/tramadol: randomised double-blind trial and confirmation of empirical theory of combination analgesics in acute pain
Source: J Headache Pain. 2015 Jun 27;16:60. doi: 10.1186/s10194-015-0541-5 (PMC4485659; doi:10.1186/s10194-015-0541-5)
Supplement: Additional file 7: — Descriptive statistics (mean and SD) for SPID, % max SPID, TOTPAR and % max TOTPAR over 4, 6, 8 and 12 h. [file 10194_2015_541_MOESM7_ESM.docx]

Additional file 7: Descriptive statistics (mean and SD) for SPID, % max SPID, TOTPAR and % max TOTPAR over 4, 6, 8 and 12 hours.

| **mean**  **(SD)** | | DKP 12.5mg + TRAM 37.5mg | DKP 12.5mg + TRAM 75mg | DKP 25mg + TRAM 37.5mg | DKP 25mg + TRAM 75mg | DKP 12.5mg | DKP 25mg | TRAM 37.5mg | TRAM 75mg | **Ibuprofen** | Placebo | Overall |
| --- | --- | --- | --- | --- | --- | --- | --- | --- | --- | --- | --- | --- |
| **SPID** |  | n=60 | n=61^a^ | n=63 | n=61 | n=60 | n=60 | n=59 | n=59 | n=60 | n=61^a^ | n=604 |
|  | 4 h | 3.8 (2.49) | 4.6 (3.24) | 4.8 (3.43) | 5.3 (3.02) | 3.3 (2.97) | 4.3  (2.94) | 1.1  (2.15) | 1.4  (2.38) | 3.3  (3.31) | 0.4 (2.05) | 3.2  (3.24) |
|  | 6 h | 4.5 (3.30) | 6.0 (4.78) | 6.4 (4.93) | 7.1 (4.54) | 3.9 (3.81) | 5.1  (4.04) | 1.4  (2.56) | 2.2  (3.47) | 4.7  (5.05) | 0.8 (3.00) | 4.2  (4.50) |
|  | 8 h | 4.8 (4.17) | 7.1 (6.11) | 7.4 (5.96) | 8.2 (5.82) | 4.3 (4.60) | 5.5  (5.03) | 1.6  (3.15) | 2.8  (4.41) | 5.9  (6.75) | 1.1 (3.77) | 4.9  (5.57) |
|  | 12 h | 5.6 (6.09) | 9.0 (8.76) | 8.6 (7.57) | 10.1 (8.41) | 4.8 (6.18) | 6.0  (6.99) | 2.4  (4.61) | 3.8  (6.33) | 7.4  (9.31) | 1.9 (5.67) | 6.0  (7.58) |
| **% max**  **SPID** |  | n=60 | n=61^a^ | n=63 | n=61 | n=60 | n=60 | n=59 | n=59 | n=60 | n=61^a^ | n=604 |
|  | 4 h | 31.4 (20.72) | 38.4 (26.99) | 40.0 (28.59) | 43.8 (25.14) | 27.4 (24.71) | 36.1 (24.48) | 8.9 (17.91) | 12.0 (19.80) | 27.7 (27.58) | 3.6 (17.09) | 27.1 (27.04) |
|  | 6 h | 24.7 (18.36) | 33.5 (26.57) | 35.7 (27.39) | 39.2 (25.22) | 21.7 (21.19) | 28.5 (22.43) | 7.5 (14.23) | 12.1 (19.26) | 26.4 (28.04) | 4.5 (16.64) | 23.5 (25.00) |
|  | 8 h | 20.1 (17.36) | 29.7 (25.45) | 30.8 (24.84) | 34.0 (24.25) | 17.8 (19.17) | 23.1 (20.94) | 6.8 (13.13) | 11.5 (18.37) | 24.6 (28.10) | 4.7 (15.70) | 20.4 (23.20) |
|  | 12 h | 15.4 (16.92) | 25.1 (24.35) | 23.9 (21.03) | 27.9 (23.37) | 13.3 (17.16) | 16.7 (19.41) | 6.6 (12.79) | 10.5 (17.58) | 20.5 (25.86) | 5.2 (15.76) | 16.6 (21.06) |
| **TOTPAR** |  | n=60 | n=62 | n=63 | n=61 | n=60 | n=60 | n=59 | n=59 | n=60 | n=62 | n=606 |
|  | 4 h | 8.6 (3.87) | 9.8 (4.56) | 9.2 (4.28) | 10.8 (3.67) | 6.6 (4.25) | 9.5  (3.73) | 3.3  (3.48) | 3.9  (4.03) | 7.5  (4.59) | 2.1 (3.16) | 7.1  (4.90) |
|  | 6 h | 10.2 (5.52) | 13.3 (7.04) | 12.6 (6.58) | 14.5 (6.14) | 7.9 (5.89) | 11.8 (5.60) | 4.0  (4.46) | 5.4  (6.10) | 10.5 (7.15) | 2.9 (4.82) | 9.3  (7.10) |
|  | 8 h | 11.2 (7.09) | 15.8 (9.56) | 14.6 (8.43) | 17.0 (8.56) | 8.8 (7.46) | 13.0 (7.17) | 4.5  (5.37) | 6.6  (8.11) | 12.8 (9.68) | 3.5 (6.37) | 10.8 (9.04) |
|  | 12 h | 12.7 (10.40) | 20.1  (14.42) | 17.2 (11.70) | 21.0 (12.96) | 10.1 (10.69) | 14.8 (10.62) | 5.6  (7.80) | 8.5 (11.68) | 15.8 (13.94) | 4.7 (9.63) | 13.1 (12.70) |
| **% max**  **TOTPAR** |  | n=60 | n=62 | n=63 | n=61 | n=60 | n=60 | n=59 | n=59 | n=60 | n=62 | n=606 |
|  | 4 h | 53.5 (24.20) | 61.1 (28.50) | 57.3 (26.78) | 67.4 (22.91) | 41.5 (26.55) | 59.3 (23.31) | 20.4 (21.77) | 24.5 (25.19) | 46.8 (28.72) | 13.3 (19.76) | 44.6 (30.62) |
|  | 6 h | 42.4 (22.99) | 55.2 (29.35) | 52.4 (27.41) | 60.5 (25.59) | 33.0 (24.55) | 49.3 (23.34) | 16.6 (18.60) | 22.5 (25.41) | 43.9 (29.81) | 12.1 (20.08) | 38.9 (29.57) |
|  | 8 h | 34.9 (22.16) | 49.4 (29.86) | 45.7 (26.35) | 53.2 (26.75) | 27.4 (23.31) | 40.5 (22.39) | 14.0 (16.77) | 20.8 (25.36) | 40.1 (30.24) | 11.0 (19.91) | 33.8 (28.25) |
|  | 12 h | 26.5 (21.66) | 42.0 (30.03) | 35.9 (24.37) | 43.7 (27.00) | 21.0 (22.28) | 30.7 (22.13) | 11.7  (16.24) | 17.7 (24.34) | 32.8 (29.04) | 9.7 (20.07) | 27.3 (26.46) |

Maximum SPID corresponds to the theoretical maximum possible time-weighted sum of the PID values, PI measured on a 4-point VRS (0=‘none’ to 3= ‘severe’). Maximum TOTPAR corresponds to the theoretical maximum possible time-weighted sum of the PAR scores, measured on a 5-point VRS (0=‘none’ to 4=‘complete’). a) One patient in the DKP12.5/TRAM75 group and one patient in the Placebo group were not included in the analysis due to missed VRS-PI assessment at baseline.
