# Supplementary material for: Dexketoprofen/tramadol: randomised double-blind trial and confirmation of empirical theory of combination analgesics in acute pain
Source: J Headache Pain. 2015 Jun 27;16:60. doi: 10.1186/s10194-015-0541-5 (PMC4485659; doi:10.1186/s10194-015-0541-5)
Supplement: Additional file 8: — Statistical Analysis of TOTPAR over 4, 6, 8 and 12 h. [file 10194_2015_541_MOESM8_ESM.docx]

Additional file 8: Statistical Analysis of TOTPAR over 4, 6, 8 and 12 hours.

| **TOTPAR** | **Treatment** | **Control** | **Estimate** | **CI Lower Limit** | **CI Upper Limit** | **Pr > \|t\|** | **Significant** |
| --- | --- | --- | --- | --- | --- | --- | --- |
| **4 h.** | **DKP12.5+Tram37.5** | **Placebo** | 6.4 | 4.5 | 8.3 | < 0.0001 | Yes |
|  | **DKP12.5+Tram75** | **Placebo** | 7.7 | 5.8 | 9.5 | < 0.0001 | Yes |
|  | **DKP25+Tram37.5** | **Placebo** | 7.0 | 5.2 | 8.9 | < 0.0001 | Yes |
|  | **DKP25+Tram75** | **Placebo** | 8.7 | 6.8 | 10.5 | < 0.0001 | Yes |
|  | **DKP12.5** | **Placebo** | 4.5 | 2.6 | 6.4 | < 0.0001 | Yes |
|  | **DKP25** | **Placebo** | 7.4 | 5.5 | 9.3 | < 0.0001 | Yes |
|  | **Tram37.5** | **Placebo** | 1.1 | -0.8 | 3.0 | 0.4718 | No |
|  | **Tram75** | **Placebo** | 1.8 | -0.1 | 3.7 | 0.0729 | No |
| **6 h.** | **DKP12.5+Tram37.5** | **Placebo** | 7.3 | 4.5 | 10.1 | < 0.0001 | Yes |
|  | **DKP12.5+Tram75** | **Placebo** | 10.4 | 7.6 | 13.2 | < 0.0001 | Yes |
|  | **DKP25+Tram37.5** | **Placebo** | 9.7 | 6.9 | 12.5 | < 0.0001 | Yes |
|  | **DKP25+Tram75** | **Placebo** | 11.6 | 8.8 | 14.4 | < 0.0001 | Yes |
|  | **DKP12.5** | **Placebo** | 5.0 | 2.2 | 7.9 | < 0.0001 | Yes |
|  | **DKP25** | **Placebo** | 8.9 | 6.1 | 11.8 | < 0.0001 | Yes |
|  | **Tram37.5** | **Placebo** | 1.1 | -1.8 | 3.9 | 0.881 | No |
|  | **Tram75** | **Placebo** | 2.5 | -0.3 | 5.3 | 0.1109 | No |
| **8 h.** | **DKP12.5+Tram37.5** | **Placebo** | 7.7 | 4.0 | 11.4 | < 0.0001 | Yes |
|  | **DKP12.5+Tram75** | **Placebo** | 12.3 | 8.6 | 16.0 | < 0.0001 | Yes |
|  | **DKP25+Tram37.5** | **Placebo** | 11.1 | 7.5 | 14.8 | < 0.0001 | Yes |
|  | **DKP25+Tram75** | **Placebo** | 13.5 | 9.8 | 17.2 | < 0.0001 | Yes |
|  | **DKP12.5** | **Placebo** | 5.3 | 1.6 | 9.0 | < 0.0001 | Yes |
|  | **DKP25** | **Placebo** | 9.5 | 5.8 | 13.2 | < 0.0001 | Yes |
|  | **Tram37.5** | **Placebo** | 1.0 | -2.7 | 4.7 | 0.9828 | No |
|  | **Tram75** | **Placebo** | 3.1 | -0.6 | 6.9 | 0.1375 | No |
| **12 h.** | **DKP12.5+Tram37.5** | **Placebo** | 8.0 | 2.6 | 13.5 | 0.0501 | No |
|  | **DKP12.5+Tram75** | **Placebo** | 15.5 | 10.1 | 20.9 | < 0.0001 | Yes |
|  | **DKP25+Tram37.5** | **Placebo** | 12.6 | 7.2 | 17.9 | < 0.0001 | Yes |
|  | **DKP25+Tram75** | **Placebo** | 16.3 | 10.9 | 21.7 | < 0.0001 | Yes |
|  | **DKP12.5** | **Placebo** | 5.4 | 0.0 | 10.9 | < 0.0001 | Yes |
|  | **DKP25** | **Placebo** | 10.1 | 4.7 | 15.5 | < 0.0001 | Yes |
|  | **Tram37.5** | **Placebo** | 1.0 | -4.5 | 6.4 | 0.9987 | No |
|  | **Tram75** | **Placebo** | 3.8 | -1.6 | 9.3 | 0.3046 | No |

PAR measured on a 5-point VRS (0=‘none’ to 4=‘complete’).
