# Supplementary material for: Dexketoprofen/tramadol: randomised double-blind trial and confirmation of empirical theory of combination analgesics in acute pain
Source: J Headache Pain. 2015 Jun 27;16:60. doi: 10.1186/s10194-015-0541-5 (PMC4485659; doi:10.1186/s10194-015-0541-5)
Supplement: Additional file 9: — Statistical analysis of percentage of max TOTPAR over 4, 6, 8 and 12 h. [file 10194_2015_541_MOESM9_ESM.docx]

Additional file 9: Statistical Analysis of percentage of max TOTPAR over 4, 6, 8 and 12 hours.

| **Percentage of max TOTPAR** | **Treatment** | **Control** | **Estimate** | **CI Lower Limit** | **CI Upper Limit** | **Pr > \|t\|** | **Significant** |
| --- | --- | --- | --- | --- | --- | --- | --- |
| **4 h.** | **DKP12.5+Tram37.5** | Placebo | 40.2 | 28.4 | 52.0 | < 0.0001 | Yes |
|  | **DKP12.5+Tram75** | Placebo | 47.8 | 36.1 | 59.5 | < 0.0001 | Yes |
|  | **DKP25+Tram37.5** | Placebo | 44.0 | 32.3 | 55.6 | < 0.0001 | Yes |
|  | **DKP25+Tram75** | Placebo | 54.2 | 42.4 | 65.9 | < 0.0001 | Yes |
|  | **DKP12.5** | Placebo | 28.3 | 16.5 | 40.1 | < 0.0001 | Yes |
|  | **DKP25** | Placebo | 46.0 | 34.2 | 57.8 | < 0.0001 | Yes |
|  | **Tram37.5** | Placebo | 7.1 | -4.7 | 19.0 | 0.4718 | No |
|  | **Tram75** | Placebo | 11.2 | -0.6 | 23.1 | 0.0729 | No |
| **6 h.** | **DKP12.5+Tram37.5** | Placebo | 30.3 | 18.5 | 42.1 | < 0.0001 | Yes |
|  | **DKP12.5+Tram75** | Placebo | 43.2 | 31.5 | 54.8 | < 0.0001 | Yes |
|  | **DKP25+Tram37.5** | Placebo | 40.4 | 28.7 | 52.0 | < 0.0001 | Yes |
|  | **DKP25+Tram75** | Placebo | 48.5 | 36.8 | 60.2 | < 0.0001 | Yes |
|  | **DKP12.5** | Placebo | 21.0 | 9.2 | 32.7 | < 0.0001 | Yes |
|  | **DKP25** | Placebo | 37.2 | 25.4 | 49.0 | < 0.0001 | Yes |
|  | **Tram37.5** | Placebo | 4.5 | -7.3 | 16.3 | 0.881 | No |
|  | **Tram75** | Placebo | 10.4 | -1.4 | 22.2 | 0.1109 | No |
| **8 h.** | **DKP12.5+Tram37.5** | Placebo | 24.0 | 12.4 | 35.5 | < 0.0001 | Yes |
|  | **DKP12.5+Tram75** | Placebo | 38.5 | 27.0 | 50.0 | < 0.0001 | Yes |
|  | **DKP25+Tram37.5** | Placebo | 34.7 | 23.3 | 46.1 | < 0.0001 | Yes |
|  | **DKP25+Tram75** | Placebo | 42.2 | 30.7 | 53.8 | < 0.0001 | Yes |
|  | **DKP12.5** | Placebo | 16.4 | 4.9 | 28.0 | < 0.0001 | Yes |
|  | **DKP25** | Placebo | 29.5 | 18.0 | 41.1 | < 0.0001 | Yes |
|  | **Tram37.5** | Placebo | 3.1 | -8.6 | 14.7 | 0.9828 | No |
|  | **Tram75** | Placebo | 9.8 | -1.8 | 21.4 | 0.1375 | No |
| **12 h.** | **DKP12.5+Tram37.5** | Placebo | 16.7 | 5.4 | 28.1 | 0.0007 | Yes |
|  | **DKP12.5+Tram75** | Placebo | 32.2 | 21.0 | 43.5 | < 0.0001 | Yes |
|  | **DKP25+Tram37.5** | Placebo | 26.2 | 15.0 | 37.3 | < 0.0001 | Yes |
|  | **DKP25+Tram75** | Placebo | 33.9 | 22.7 | 45.2 | < 0.0001 | Yes |
|  | **DKP12.5** | Placebo | 11.3 | 0.0 | 22.6 | 0.0501 | No |
|  | **DKP25** | Placebo | 21.0 | 9.7 | 32.3 | < 0.0001 | Yes |
|  | **Tram37.5** | Placebo | 2.0 | -9.3 | 13.4 | 0.9987 | No |
|  | **Tram75** | Placebo | 7.9 | -3.4 | 19.3 | 0.3046 | No |

Maximum TOTPAR corresponds to the theoretical maximum possible time-weighted sum of the PAR scores, measured on a 5-point VRS (0=‘none’ to 4=‘complete’).
