# Supplementary material for: Dexketoprofen/tramadol: randomised double-blind trial and confirmation of empirical theory of combination analgesics in acute pain
Source: J Headache Pain. 2015 Jun 27;16:60. doi: 10.1186/s10194-015-0541-5 (PMC4485659; doi:10.1186/s10194-015-0541-5)
Supplement: Additional file 10: — Statistical analysis of SPID over 4, 6, 8 and 12 h. [file 10194_2015_541_MOESM10_ESM.docx]

Additional file 10: Statistical Analysis of SPID over 4, 6, 8 and 12 hours.

| **SPID** | **Treatment** | **Control** | **Estimate** | **CI Lower Limit** | **CI Upper Limit** | **Pr > \|t\|** | **Significant** |
| --- | --- | --- | --- | --- | --- | --- | --- |
| **4 h.** | **DKP12.5+Tram37.5** | **Placebo** | 3.6 | 2.4 | 4.9 | < 0.0001 | Yes |
|  | **DKP12.5+Tram75** | **Placebo** | 4.5 | 3.3 | 5.7 | < 0.0001 | Yes |
|  | **DKP25+Tram37.5** | **Placebo** | 4.5 | 3.3 | 5.7 | < 0.0001 | Yes |
|  | **DKP25+Tram75** | **Placebo** | 5.1 | 3.9 | 6.3 | < 0.0001 | Yes |
|  | **DKP12.5** | **Placebo** | 2.9 | 1.7 | 4.1 | < 0.0001 | Yes |
|  | **DKP25** | **Placebo** | 4.3 | 3.0 | 5.5 | < 0.0001 | Yes |
|  | **Tram37.5** | **Placebo** | 0.9 | -0.3 | 2.1 | 0.2697 | No |
|  | **Tram75** | **Placebo** | 1.1 | -0.1 | 2.4 | 0.0808 | No |
| **6 h.** | **DKP12.5+Tram37.5** | **Placebo** | 4.0 | 2.3 | 5.8 | < 0.0001 | Yes |
|  | **DKP12.5+Tram75** | **Placebo** | 5.6 | 3.9 | 7.4 | < 0.0001 | Yes |
|  | **DKP25+Tram37.5** | **Placebo** | 5.8 | 4.0 | 7.5 | < 0.0001 | Yes |
|  | **DKP25+Tram75** | **Placebo** | 6.6 | 4.8 | 8.3 | < 0.0001 | Yes |
|  | **DKP12.5** | **Placebo** | 3.1 | 1.4 | 4.9 | < 0.0001 | Yes |
|  | **DKP25** | **Placebo** | 4.8 | 3.0 | 6.5 | < 0.0001 | Yes |
|  | **Tram37.5** | **Placebo** | 0.9 | -0.9 | 2.6 | 0.6641 | No |
|  | **Tram75** | **Placebo** | 1.5 | -0.2 | 3.3 | 0.1068 | No |
| **8 h.** | **DKP12.5+Tram37.5** | **Placebo** | 4.1 | 1.9 | 6.4 | < 0.0001 | Yes |
|  | **DKP12.5+Tram75** | **Placebo** | 6.5 | 4.3 | 8.7 | < 0.0001 | Yes |
|  | **DKP25+Tram37.5** | **Placebo** | 6.4 | 4.2 | 8.6 | < 0.0001 | Yes |
|  | **DKP25+Tram75** | **Placebo** | 7.4 | 5.2 | 9.6 | < 0.0001 | Yes |
|  | **DKP12.5** | **Placebo** | 3.2 | 1.0 | 5.4 | 0.0011 | Yes |
|  | **DKP25** | **Placebo** | 4.9 | 2.7 | 7.1 | < 0.0001 | Yes |
|  | **Tram37.5** | **Placebo** | 0.9 | -1.3 | 3.1 | 0.8628 | No |
|  | **Tram75** | **Placebo** | 1.8 | -0.4 | 4.0 | 0.1569 | No |
| **12 h.** | **DKP12.5+Tram37.5** | **Placebo** | 4.3 | 1.1 | 7.4 | 0.0028 | Yes |
|  | **DKP12.5+Tram75** | **Placebo** | 7.8 | 4.6 | 10.9 | < 0.0001 | Yes |
|  | **DKP25+Tram37.5** | **Placebo** | 6.9 | 3.8 | 10.1 | < 0.0001 | Yes |
|  | **DKP25+Tram75** | **Placebo** | 8.6 | 5.5 | 11.8 | < 0.0001 | Yes |
|  | **DKP12.5** | **Placebo** | 3.0 | -0.2 | 6.2 | 0.072 | No |
|  | **DKP25** | **Placebo** | 4.8 | 1.6 | 7.9 | 0.0005 | Yes |
|  | **Tram37.5** | **Placebo** | 1.0 | -2.2 | 4.1 | 0.9596 | No |
|  | **Tram75** | **Placebo** | 2.2 | -1.0 | 5.3 | 0.3329 | No |

PI measured on a 4-point VRS (0=‘none’ to 3= ‘severe’).
