# Supplementary material for: Dexketoprofen/tramadol: randomised double-blind trial and confirmation of empirical theory of combination analgesics in acute pain
Source: J Headache Pain. 2015 Jun 27;16:60. doi: 10.1186/s10194-015-0541-5 (PMC4485659; doi:10.1186/s10194-015-0541-5)
Supplement: Additional file 11: — Statistical analysis of percentage of max SPID over 4, 6, 8 and 12 h. [file 10194_2015_541_MOESM11_ESM.docx]

Additional file 11: Statistical Analysis of percentage of max SPID over 4, 6, 8 and 12 hours.

| **Percentage of max SPID** | **Treatment** | **Control** | **Estimate** | **CI Lower Limit** | **CI Upper Limit** | **Pr > \|t\|** | **Significant** |
| --- | --- | --- | --- | --- | --- | --- | --- |
| **4 h.** | **DKP12.5+Tram37.5** | **Placebo** | 30.4 | 20.2 | 40.6 | < 0.0001 | Yes |
|  | **DKP12.5+Tram75** | **Placebo** | 37.5 | 27.3 | 47.6 | < 0.0001 | Yes |
|  | **DKP25+Tram37.5** | **Placebo** | 37.3 | 27.3 | 47.4 | < 0.0001 | Yes |
|  | **DKP25+Tram75** | **Placebo** | 42.2 | 32.1 | 52.4 | < 0.0001 | Yes |
|  | **DKP12.5** | **Placebo** | 24.0 | 13.9 | 34.2 | < 0.0001 | Yes |
|  | **DKP25** | **Placebo** | 35.4 | 25.2 | 45.6 | < 0.0001 | Yes |
|  | **Tram37.5** | **Placebo** | 7.4 | -2.8 | 17.6 | 0.2697 | No |
|  | **Tram75** | **Placebo** | 9.5 | -0.7 | 19.7 | 0.0808 | No |
| **6 h.** | **DKP12.5+Tram37.5** | **Placebo** | 22.5 | 12.8 | 32.2 | < 0.0001 | Yes |
|  | **DKP12.5+Tram75** | **Placebo** | 31.4 | 21.8 | 41.0 | < 0.0001 | Yes |
|  | **DKP25+Tram37.5** | **Placebo** | 32.0 | 22.5 | 41.6 | < 0.0001 | Yes |
|  | **DKP25+Tram75** | **Placebo** | 36.5 | 26.8 | 46.1 | < 0.0001 | Yes |
|  | **DKP12.5** | **Placebo** | 17.4 | 7.7 | 27.0 | < 0.0001 | Yes |
|  | **DKP25** | **Placebo** | 26.6 | 16.9 | 36.2 | < 0.0001 | Yes |
|  | **Tram37.5** | **Placebo** | 4.9 | -4.8 | 14.6 | 0.6641 | No |
|  | **Tram75** | **Placebo** | 8.6 | -1.1 | 18.3 | 0.1068 | No |
| **8 h.** | **DKP12.5+Tram37.5** | **Placebo** | 17.3 | 8.1 | 26.5 | < 0.0001 | Yes |
|  | **DKP12.5+Tram75** | **Placebo** | 27.0 | 17.8 | 36.2 | < 0.0001 | Yes |
|  | **DKP25+Tram37.5** | **Placebo** | 26.7 | 17.6 | 35.8 | < 0.0001 | Yes |
|  | **DKP25+Tram75** | **Placebo** | 30.8 | 21.6 | 39.9 | < 0.0001 | Yes |
|  | **DKP12.5** | **Placebo** | 13.2 | 4.0 | 22.4 | 0.0011 | Yes |
|  | **DKP25** | **Placebo** | 20.5 | 11.3 | 29.7 | < 0.0001 | Yes |
|  | **Tram37.5** | **Placebo** | 3.6 | -5.6 | 12.9 | 0.8628 | No |
|  | **Tram75** | **Placebo** | 7.6 | -1.6 | 16.8 | 0.1569 | No |
| **12 h.** | **DKP12.5+Tram37.5** | **Placebo** | 11.8 | 3.0 | 20.6 | 0.0028 | Yes |
|  | **DKP12.5+Tram75** | **Placebo** | 21.6 | 12.8 | 30.3 | < 0.0001 | Yes |
|  | **DKP25+Tram37.5** | **Placebo** | 19.3 | 10.6 | 27.9 | < 0.0001 | Yes |
|  | **DKP25+Tram75** | **Placebo** | 24.0 | 15.2 | 32.7 | < 0.0001 | Yes |
|  | **DKP12.5** | **Placebo** | 8.3 | -0.5 | 17.1 | 0.072 | No |
|  | **DKP25** | **Placebo** | 13.3 | 4.5 | 22.1 | 0.0005 | Yes |
|  | **Tram37.5** | **Placebo** | 2.7 | -6.1 | 11.5 | 0.9596 | No |
|  | **Tram75** | **Placebo** | 6.0 | -2.8 | 14.8 | 0.3329 | No |

Maximum SPID corresponds to the theoretical maximum possible time-weighted sum of the PID values, PI measured on a 4-point VRS (0=‘none’ to 3= ‘severe’).
