# Supplementary material for: Dexketoprofen/tramadol: randomised double-blind trial and confirmation of empirical theory of combination analgesics in acute pain
Source: J Headache Pain. 2015 Jun 27;16:60. doi: 10.1186/s10194-015-0541-5 (PMC4485659; doi:10.1186/s10194-015-0541-5)
Supplement: Additional file 15: — Time to RM (Kaplan-Meier estimation) over 24 h. [file 10194_2015_541_MOESM15_ESM.docx]

Additional file 15: Time to RM (Kaplan-Meier estimation) over 24hours.

|  | | DKP 12.5mg + TRAM 37.5mg | DKP 12.5mg + TRAM 75mg | DKP 25mg + TRAM 37.5mg | DKP 25mg + TRAM 75mg | DKP 12.5mg | DKP 25mg | TRAM 37.5mg | TRAM 75mg | Ibuprofen | Placebo |
| --- | --- | --- | --- | --- | --- | --- | --- | --- | --- | --- | --- |
|  |  | n=60 | n=62 | n=63 | n=61 | n=60 | n=60 | n=59 | n=59 | n=60 | n=62 |
| **Estimated time to RM (hours)** | **median (95% CI)** | 4.9 ^a^  (4.0,5.8) | 8.5 ^a^  (5.9,13.0) | 7.3 ^a^  (6.3,9.0) | 8.1 ^a^  (6.3,13.4) | 3.6 ^b^  (2.7,4.3) | 5.6 ^a^  (4.8,7.6) | 2.2  (1.3,3.0) | 2.5  (1.4,3.9) | 7.1 ^a^  (4.8,8.6) | 1.4  (1.2,1.8) |

a) P<0.0001 vs. placebo; b) P=0.0037 vs. placebo; RM: rescue medication; time to RM is defined as the time elapsed between the treatment administration and the first RM use; the survival distribution estimates the probability of ‘no use of RM’ at each time point.
