# Supplementary material for: Dexketoprofen/tramadol: randomised double-blind trial and confirmation of empirical theory of combination analgesics in acute pain
Source: J Headache Pain. 2015 Jun 27;16:60. doi: 10.1186/s10194-015-0541-5 (PMC4485659; doi:10.1186/s10194-015-0541-5)
Supplement: Additional file 16: — Cumulative percentage of patients who required RM over 4, 6, 8, 12 and 24 h. [file 10194_2015_541_MOESM16_ESM.docx]

Additional file 16: Cumulative percentage of patients who required RM over 4, 6, 8, 12 and 24 hours.

|  | | DKP 12.5mg + TRAM 37.5mg | DKP 12.5mg + TRAM 75mg | DKP 25mg + TRAM 37.5mg | DKP 25mg + TRAM 75mg | DKP 12.5mg | DKP 25mg | TRAM 37.5mg | TRAM 75mg | Ibuprofen | Placebo | Overall |
| --- | --- | --- | --- | --- | --- | --- | --- | --- | --- | --- | --- | --- |
|  |  | n=60 | n=62 | n=63 | n=61 | n=60 | n=60 | n=59 | n=59 | n=60 | n=62 | n=606 |
| **RM**  **at 4 h** | n  (%) | 29  (48.3) | 19  (30.6) **^a^** | 12  (19.0) **^a^** | 9  (14.8) **^a^** | 34  (56.7) | 20  (33.3) **^a^** | 40  (67.8) | 35  (59.3) | 21  (35.0) | 43  (69.4) | 262 (43.2) |
| **RM**  **at 6 h** | n  (%) | 40  (66.7) | 29  (46.8) **^c^** | 25  (39.7) **^b^** | 23  (37.7) **^a^** | 39  (65.0) | 32  (53.3) | 41  (69.5) | 38  (64.4) | 29  (48.3) | 45  (72.6) | 341 (56.3) |
| **RM**  **at 8 h** | n  (%) | 43  (71.7) | 33  (53.2) | 31  (49.2) | 29  (47.5) **^c^** | 39  (65.0) | 36  (60.0) | 41  (69.5) | 38  (64.4) | 34  (56.7) | 45  (72.6) | 369 (60.9) |
| **RM**  **at 12 h** | n  (%) | 45  (75.0) | 36  (58.1) | 31  (49.2) **^c^** | 33  (54.1) | 39  (65.0) | 37  (61.7) | 43  (72.9) | 39  (66.1) | 39  (65.0) | 46  (74.2) | 388 (64.0) |
| **RM**  **at 24 h** | n  (%) | 45  (75.0) | 36  (58.1) | 31  (49.2) **^c^** | 33  (54.1) | 39  (65.0) | 37  (61.7) | 43  (72.9) | 39  (66.1) | 39  (65.0) | 46  (74.2) | 388 (64.0) |

a) P≤0.0001 vs. placebo; b) P=0.0003 vs. placebo; c) P<0.00639 vs. placebo; RM: rescue medication.
