# Supplementary material for: Dexketoprofen/tramadol: randomised double-blind trial and confirmation of empirical theory of combination analgesics in acute pain
Source: J Headache Pain. 2015 Jun 27;16:60. doi: 10.1186/s10194-015-0541-5 (PMC4485659; doi:10.1186/s10194-015-0541-5)
Supplement: Additional file 17: — Statistical analysis of percentage of patients who required RM over 4, 6, 8, 12 and 24 h. [file 10194_2015_541_MOESM17_ESM.docx]

Additional file 17: Statistical Analysis of the percentage of patients who required RM over 4, 6, 8, 12 and 24 hours.

| **Rescue medication** | **Treatment** | **Control** | **Estimate** | **Standard Error** | **CI Lower Limit** | **CI Upper Limit** | **Pr > \|z\|** | **Significant** |
| --- | --- | --- | --- | --- | --- | --- | --- | --- |
| **4 hours** | **DKP12.5+Tram37.5** | **Placebo** | -0.88 | 0.38 | -1.91 | 0.15 | 0.01932 | No |
|  | **DKP12.5+Tram75** | **Placebo** | -1.63 | 0.39 | -2.70 | -0.57 | 0.00003 | Yes |
|  | **DKP25+Tram37.5** | **Placebo** | -2.26 | 0.42 | -3.42 | -1.11 | < 0.0001 | Yes |
|  | **DKP25+Tram75** | **Placebo** | -2.57 | 0.45 | -3.81 | -1.33 | < 0.0001 | Yes |
|  | **DKP12.5** | **Placebo** | -0.55 | 0.38 | -1.58 | 0.49 | 0.148 | No |
|  | **DKP25** | **Placebo** | -1.51 | 0.39 | -2.57 | -0.45 | 0.0001 | Yes |
|  | **Tram37.5** | **Placebo** | -0.07 | 0.39 | -1.14 | 1.0 | 0.85356 | No |
|  | **Tram75** | **Placebo** | -0.44 | 0.38 | -1.48 | 0.60 | 0.25029 | No |
| **6 hours** | **DKP12.5+Tram37.5** | **Placebo** | -0.28 | 0.40 | -1.36 | 0.80 | 0.47797 | No |
|  | **DKP12.5+Tram75** | **Placebo** | -1.10 | 0.38 | -2.14 | -0.06 | 0.00388 | Yes |
|  | **DKP25+Tram37.5** | **Placebo** | -1.39 | 0.38 | -2.44 | -0.35 | 0.00029 | Yes |
|  | **DKP25+Tram75** | **Placebo** | -1.48 | 0.39 | -2.53 | -0.42 | 0.00015 | Yes |
|  | **DKP12.5** | **Placebo** | -0.35 | 0.39 | -1.43 | 0.72 | 0.36694 | No |
|  | **DKP25** | **Placebo** | -0.84 | 0.38 | -1.89 | 0.21 | 0.02902 | No |
|  | **Tram37.5** | **Placebo** | -0.15 | 0.40 | -1.24 | 0.94 | 0.70806 | No |
|  | **Tram75** | **Placebo** | -0.38 | 0.39 | -1.45 | 0.69 | 0.33392 | No |
| **8 hours** | **DKP12.5+Tram37.5** | **Placebo** | -0.05 | 0.40 | -1.15 | 1.06 | 0.91038 | No |
|  | **DKP12.5+Tram75** | **Placebo** | -0.84 | 0.38 | -1.89 | 0.20 | 0.02705 | No |
|  | **DKP25+Tram37.5** | **Placebo** | -1.01 | 0.38 | -2.04 | 0.03 | 0.0082 | No |
|  | **DKP25+Tram75** | **Placebo** | -1.07 | 0.38 | -2.12 | -0.03 | 0.00514 | Yes |
|  | **DKP12.5** | **Placebo** | -0.35 | 0.39 | -1.43 | 0.72 | 0.36694 | No |
|  | **DKP25** | **Placebo** | -0.57 | 0.39 | -1.63 | 0.49 | 0.14316 | No |
|  | **Tram37.5** | **Placebo** | -0.15 | 0.40 | -1.24 | 0.94 | 0.70806 | No |
|  | **Tram75** | **Placebo** | -0.38 | 0.39 | -1.45 | 0.69 | 0.33392 | No |
| **12 hours** | **DKP12.5+Tram37.5** | **Placebo** | 0.04 | 0.42 | -1.09 | 1.18 | 0.91853 | No |
|  | **DKP12.5+Tram75** | **Placebo** | -0.73 | 0.39 | -1.79 | 0.33 | 0.05964 | No |
|  | **DKP25+Tram37.5** | **Placebo** | -1.09 | 0.38 | -2.14 | -0.04 | 0.00465 | Yes |
|  | **DKP25+Tram75** | **Placebo** | -0.89 | 0.39 | -1.95 | 0.17 | 0.02142 | No |
|  | **DKP12.5** | **Placebo** | -0.44 | 0.40 | -1.52 | 0.65 | 0.27082 | No |
|  | **DKP25** | **Placebo** | -0.58 | 0.39 | -1.65 | 0.49 | 0.13994 | No |
|  | **Tram37.5** | **Placebo** | -0.07 | 0.41 | -1.19 | 1.06 | 0.87007 | No |
|  | **Tram75** | **Placebo** | -0.39 | 0.40 | -1.48 | 0.70 | 0.33159 | No |
| **24 hours** | **DKP12.5+Tram37.5** | **Placebo** | 0.04 | 0.42 | -1.09 | 1.18 | 0.91853 | No |
|  | **DKP12.5+Tram75** | **Placebo** | -0.73 | 0.39 | -1.79 | 0.33 | 0.05964 | No |
|  | **DKP25+Tram37.5** | **Placebo** | -1.09 | 0.38 | -2.14 | -0.04 | 0.00465 | Yes |
|  | **DKP25+Tram75** | **Placebo** | -0.89 | 0.39 | -1.95 | 0.17 | 0.02142 | No |
|  | **DKP12.5** | **Placebo** | -0.44 | 0.40 | -1.52 | 0.65 | 0.27082 | No |
|  | **DKP25** | **Placebo** | -0.58 | 0.39 | -1.65 | 0.49 | 0.13994 | No |
|  | **Tram37.5** | **Placebo** | -0.07 | 0.41 | -1.19 | 1.06 | 0.87007 | No |
|  | **Tram75** | **Placebo** | -0.39 | 0.40 | -1.48 | 0.70 | 0.33159 | No |
